# Acetylation of lysine 49 on Ctnnb1 drives naïve pluripotency in murine stem cells by modulating Nanog function

**DOI:** 10.1093/pnasnexus/pgaf297

**Published:** 2025-09-12

**Authors:** Toshiyuki Takehara, Mahito Nakanishi, Raku Son, Hirofumi Suemori, Yasuhiro Murakawa, Takeshi Teramura

**Affiliations:** Division of Cell Biology for Regenerative Medicine, Institute of Advanced Clinical Medicine, Kindai University Hospital, 377-2 Onohigashi, Osaka-sayama, Osaka 589-8511, Japan; Tokiwa Bio Inc., Tsukuba Center Inc., Bulding G, 2-1-6 Sengen, Tsukuba-shi, Ibaraki 305-0047, Japan; Research Center for Stem Cell Engineering, National Institute of Advanced Industrial Science and Technology, 1-1-1 Umezono, Tsukuba, Ibaraki 305-8560, Japan; RIKEN-IFOM Joint Laboratory for Cancer Genomics, RIKEN Center for Integrative Medical Sciences, 1-7-22, Suehiro-cho, Tsurumi-ku, Yokohama, Kanagawa 230-8611, Japan; Institute for the Advanced Study of Human Biology, Kyoto University, Yoshida-Konoe-cho, Sakyo-ku, Kyoto 606-8501, Japan; Department of Nephrology, Graduate School of Medicine, Kyoto University, Yoshida-Konoe-cho, Sakyo-ku, Kyoto 606-8501, Japan; Laboratory of Embryonic Stem Cell Research, Institute for Frontier Life and Medical Sciences, Kyoto University, Shogoin Kawahara-cho, Sakyo-ku, Kyoto 606-8507, Japan; RIKEN-IFOM Joint Laboratory for Cancer Genomics, RIKEN Center for Integrative Medical Sciences, 1-7-22, Suehiro-cho, Tsurumi-ku, Yokohama, Kanagawa 230-8611, Japan; Institute for the Advanced Study of Human Biology, Kyoto University, Yoshida-Konoe-cho, Sakyo-ku, Kyoto 606-8501, Japan; IFOM ETS-the AIRC Institute of Molecular Oncology, Via Adamello, 16, 20139 Milano, Italy; Division of Cell Biology for Regenerative Medicine, Institute of Advanced Clinical Medicine, Kindai University Hospital, 377-2 Onohigashi, Osaka-sayama, Osaka 589-8511, Japan

**Keywords:** pluripotency, epiblast stem cells, Ctnnb1, Crebbp/Ep300, posttranslational modification

## Abstract

Naïve pluripotency represents the ground state of mammalian development. A comprehensive understanding of the molecular mechanisms governing its establishment is crucial for elucidating the unique properties of embryonic cells and the regulatory mechanisms controlling cell fate determination. However, the key molecule to robustly achieve naïve pluripotency with minimal manipulation remains unclear. We found that the acetylation status of lysine 49 (K49) of Catenin beta-1 (Ctnnb1) plays a critical role in naïve pluripotency of murine stem cells. Deacetylated Ctnnb1 at K49 binds to transcription factor Nanog, impeding its repressor function and thereby promoting differentiation. Remarkably, treatment with IQ1, an inhibitor of interaction between acetyltransferase Ep300 and Ctnnb1, enhances acetylation at K49 of Ctnnb1, enabling the establishment and long-term maintenance of embryonic stem cells independently of the leukemia inhibitory factor, and also driving complete conversion of epiblast stem cells to the naïve state. This study reveals the critical role of Ctnnb1 in naïve pluripotency and introduces an effective strategy for its induction and maintenance.

Significance Statement Ctnnb1^K49^ is acetylated specifically in the naïve pluripotent state. We demonstrated that this modification is important for the suppression of differentiation-associated gene expression by Nanog. Upon differentiation, acetylation at K49 of Ctnnb1 is diminished. Notably, the overexpression of a constitutively K49 acetylated Ctnnb1 mutant or treatment with IQ1, which inhibits the interaction between Ctnnb1 and Ep300, enabled the establishment and long-term maintenance of embryonic stem cells independent of the leukemia inhibitory factor. Furthermore, IQ1 treatment induced a fully naïve pluripotent state in epiblast stem cells, which contributed to chimeric mice following blastocyst injection. These findings demonstrate that K49 acetylation of Ctnnb1 plays a crucial role in the establishment of naïve pluripotency.

## Introduction

In mammals, pluripotent stem cells arise during the blastocyst stage. After implantation, their differentiation pathways become progressively restricted, becoming founder cells committed to specific lineages. In mice, at least three types of stem cells have been used to model early embryogenesis: embryonic stem cells (ESCs), representing preimplantation pluripotency; epiblast stem cells (EpiSCs), representing postimplantation pluripotency at E4.5–7.5 (E, days post coitus), and formative stem cells representing transitional stages between ESCs and EpiSCs (1, 2).

Naïve and primed state stem cells use different molecular pathways to maintain self-renewal. In the former, self-renewal is sustained by the leukemia inhibitory factor (LIF)-Stat3 pathway, while Fgf2/Erk signaling promotes early differentiation. In contrast, in the latter state, the LIF-Stat3 pathway is ineffective, with self-renewal being supported by the Fgf2-Erk and Activin-Smad signaling cascades. During the transition from naïve to primed states, the expression of numerous signaling molecules and transcription factors quantitatively fluctuates through transcriptional regulation and/or posttranslational degradation. However, key transcription factors, including Pou5f1, Sox2, and Nanog, as well as signal mediators including Erk, are not merely switched on or off, but instead exhibit multifunctional roles across multiple pluripotent states (3, 4).

Ctnnb1 is a major protein present in both the naïve and the primed states, with versatile functions in regulating pluripotency (1, 3). Its general functions include regulating cell adhesion through binding to cadherins (5), facilitating cell migration by controlling myosin activation (6), and serving as a central mediator in canonical Wnt signaling (7–10). In pluripotent stem cells, it has been demonstrated that stabilization and increased abundance of Ctnnb1 contribute to the maintenance of the naïve state pluripotency (11–14). By contrast, in the primed state, Ctnnb1 does not appear to positively contribute to the maintenance of self-renewal; instead, it is hypothesized to promote differentiation (15–17). During activation of canonical Wnt/Ctnnb1 signaling, Ctnnb1 interacts with Tcf and Lef, functioning as a transcriptional regulator to activate the transcription of the target genes (10, 18). In naïve-state stem cells, inhibiting the interaction between Ctnnb1 and Tcf using iCRT3 enhances pluripotency (19). Therefore, both Wnt/Ctnnb1 activity and Tcf-mediated transcription appear to play a fundamental role in initiating differentiation. This hypothesis is supported by the demonstration that Ctnnb1/Tcf-dependent transcriptional activation is relatively low in the naïve state, but increases during murine ESC differentiation (19, 20). On the other hand, the molecular mechanisms by which Ctnnb1 supports naïve pluripotency but not in primed state stem cells remain unclear.

We demonstrate that acetylation of Ctnnb1 at lysine 49 (K49) plays a critical role in regulating pluripotency in murine naïve-state stem cells through its interaction with Nanog. Furthermore, we show that the naïve pluripotent state can be induced and maintained in a LIF-independent manner by treatment with IQ1, which enhances K49 acetylation of Ctnnb1. These findings highlight the essential role of Ctnnb1 acetylation in the molecular regulation of pluripotency.

## Results

### Ctnnb1 K49 is highly acetylated in murine naïve-state stem cells

**Fig. 1.**
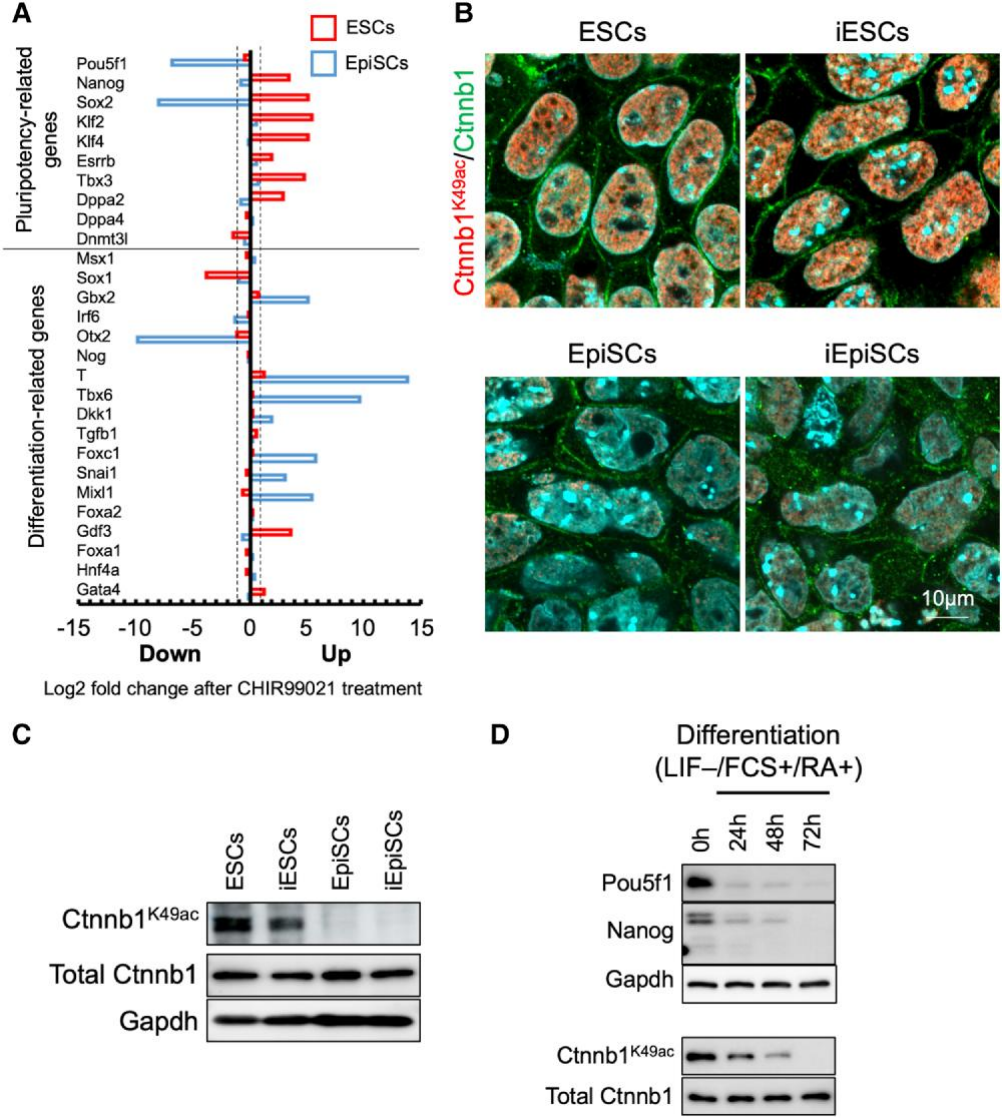
Mouse naïve state stem cells exhibit increased K49 acetylation of Ctnnb1. A) Changes in pluripotency- and differentiation-associated gene expression in response to increased Ctnnb1 levels in ESCs (naïve) and EpiSCs (primed). Signature genes associated with pluripotency and early differentiation were identified from microarray data and are presented. B) Subcellular localization and abundance of Ctnnb1^K49ac^ in ESCs and EpiSCs. Ctnnb1^K49ac^ was visualized using CF555, total Ctnnb1 with CF488, and DNA with DAPI. C) WB analysis of Ctnnb1^K49ac^ expression in naïve and primed state stem cells, including detection of total Ctnnb1. D) WB confirming the reduction of Pou5f1, Nanog, and Ctnnb1^K49ac^ by differentiation induction.

To investigate the role of Ctnnb1 in pluripotency, we treated mouse ESCs and EpiSCs with CHIR99021 and analyzed gene expression. In ESCs, this treatment increased the expression of pluripotency-related genes. In contrast, CHIR99021 treatment increased the expression of differentiation-related genes in EpiSCs (Fig. 1A).

As a potential mechanism underlying this different gene expression response, we focused on the acetylation of K49 residue on Ctnnb1. Immunofluorescence revealed that K49-acetylated Ctnnb1 (Ctnnb1^K49ac^) was localized to the nuclei of undifferentiated mouse ESCs (Figs. 1B and S1). To determine whether Ctnnb1^K49ac^ is specifically associated with the naïve state rather than being exclusive to ESCs, we measured the abundance of Ctnnb1^K49ac^ by western blotting (WB) in two different naïve-state stem cell lines (ESCs and ES-like cells derived from EpiSCs by treatment with A83-01, PD0325901, and CHIR99021 [induced ESCs; iESCs]) versus two primed-state stem-cell lines (EpiSCs and EpiSC-like cells derived from ESCs by Fgf2 stimulation in the absence of LIF [induced EpiSCs; iEpiSCs] (21)). Ctnnb1^K49ac^ was significantly abundant in the naïve state than in the primed state. By contrast, total Ctnnb1 expression did not differ across the four stem-cell types (Fig. 1C). In ESCs, Ctnnb1^K49ac^ decreased upon differentiation induction by LIF withdrawal, addition of fetal calf serum, and retinoic acid. At 72 h postdifferentiation induction, Ctnnb1^K49ac^ was nearly undetectable; moreover, the expression of Pou5f1 and Nanog also declined (Fig. 1D).

### Acetylation of K49 on Ctnnb1 alters its affinity for interaction with Nanog and modulates the repressor function of Nanog

**Fig. 2.**
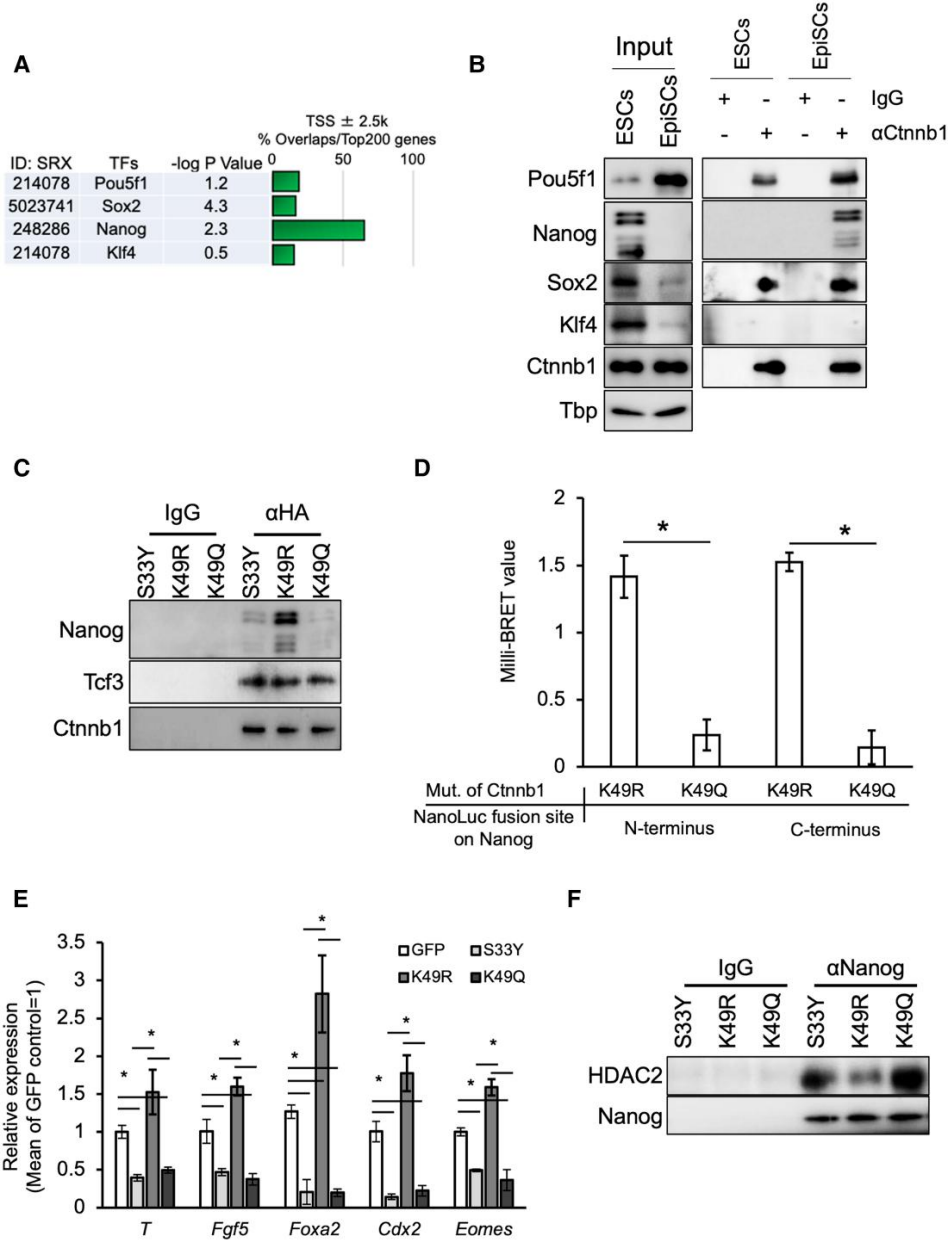
Ctnnb1^K49ac^ affects its binding with Nanog and regulates Nanog function as a repressor of differentiation-related gene expression. A) In silico ChIP analysis of transcription factor occupancy in the promoter regions of genes whose expression is Ctnnb1-dependent, showing enrichment of Nanog binding sites. B) IP confirming the interaction between Ctnnb1 and pluripotency-regulating transcription factors Pou5f1, Nanog, Sox2, and Klf4. C) Verification of binding between each Ctnnb1 mutant and Nanog or Tcf3. The nonacetylated mimic K49R exhibits strong binding to Nanog. D) Quantitative analysis of the interaction between Ctnnb1^K49R^ or Ctnnb1^K49Q^ and Nanog using NanoBRET. HaloTag was fused to the N-terminus of each Ctnnb1 variant, and interaction with NanoLuc-fused Nanog was assessed. The bars represent the mean ± SD of three biological replicates (*n* = 3). The asterisks indicate statistically significant differences between groups (*P* < 0.05). E) Expression changes in differentiation-related/primed-state signature genes upon the forced expression of each Ctnnb1 mutant in ESCs. Ctnnb1^S33Y^(S33Y) is a stabilized mutant form of Ctnnb1 that served as the parental construct for the Ctnnb1^K49R^ and Ctnnb1^K49Q^ mutants, and was used as a control in this experiment. The bars represent the mean ± SD of three biological replicates (*n* = 3). The asterisks indicate statistically significant differences between groups (*P* < 0.05). F) IP demonstrating changes in interaction between Nanog and HDAC2 in ESCs following the forced expression of each Ctnnb1 mutant.

Based on the differences in the abundance of Ctnnb1^K49ac^ in the two pluripotent cell types, we hypothesized that Ctnnb1^K49ac^ contributes to the unique characteristics of each stem cell type by interacting with core transcription factors. To test this, we used in silico analysis to examine the promoters of genes whose expression was altered by CHIR99021 treatment in ESCs and EpiSCs, specifically binding of the core transcription factors Pou5f1, Sox2, Nanog, and Klf4, and found abundant Nanog binding on the promoter regions (Fig. 2A). To test for a physical interaction between Ctnnb1 and Nanog, we conducted immunoprecipitation (IP) assays on nuclear lysates from ESCs and EpiSCs using anti-Pou5f1, Nanog, Sox2, and Klf4 antibodies. While these assays suggest that Ctnnb1 interacts with Pou5f1 and Sox2 in both ESCs and EpiSCs, the interaction between Ctnnb1 and Nanog was detected only in EpiSCs (Fig. 2B).

In silico protein–protein interaction prediction between Ctnnb1 and Nanog based on the AlphaFold Protein Structure Database and docking simulations with HADDOCK (22, 23) revealed that the buried surface area between Ctnnb1 and Nanog was 2,889.345 Å^2^ ± 92.301 in the K49 deacetylated Ctnnb1 model, whereas it decreased to 2,680.945 Å^2^ ± 139.438 in the K49 acetylated model. The electrostatic energy was −364.2 ± 98.3 kcal/mol in the K49 deacetylated Ctnnb1–Nanog complex and −182.0 ± 31.9 kcal/mol in the K49-acetylated complex. These results suggest that K49 acetylation at K49 weakens the interaction between Ctnnb1 and Nanog. To further investigate the impact of acetylation at the K49 residue of Ctnnb1 on interaction between Ctnnb1 and Nanog, we generated two mutants based on the stabilized form of Ctnnb1 (Ctnnb1^S33Y^): Ctnnb1^K49R^, which mimics the deacetylated state by substituting lysine with arginine, and Ctnnb1^K49Q^, which mimics the acetylated state by substituting with glutamine. These constructs were ectopically expressed in ESCs, followed by IP. The acetylation-mimic mutant Ctnnb1^K49Q^, as well as the parental Ctnnb1^S33Y^ construct, showed only weak or negligible interaction with Nanog. In contrast, the deacetylation-mimic Ctnnb1^K49R^ exhibited strong binding to Nanog. Notably, the interaction with Tcf3, a canonical binding partner of Ctnnb1 in pluripotency, was unaffected across all three variants (Fig. 2C). Then, we performed a bioluminescence resonance energy transfer (BRET) assay using Ctnnb1 mutants to mimic its deacetylated and acetylated states to confirm that K49 acetylation on Ctnnb1 inhibits interaction between Ctnnb1 and Nanog. Ctnnb1^K49R^ and Ctnnb1^K49Q^ were each fused at the N-terminus with HaloTag and coexpressed in HEK293 cells with Nanog fused to NanoLuc at either its N- or C-terminus. The NanoBRET assay revealed that the interaction signal between Ctnnb1 and Nanog observed with the Ctnnb1^K49R^ mutant was significantly reduced in the Ctnnb1^K49Q^ mutant (Fig. 2D).

To investigate the effect of Ctnnb1^K49ac^ on Nanog-dependent transcription regulation, ESCs were transfected with plasmids expressing all three mutants, followed by measuring the expression of early differentiation–associated genes. RT-PCR showed that while Ctnnb1^S33Y^ and Ctnnb1^K49Q^ significantly repressed the expression of the early differentiation genes *T*, *Fgf5*, *Foxa2*, *Cdx2*, and *Eomes*, all are typically suppressed by Nanog in naïve pluripotency. Contrarily, these genes are up-regulated in the ESCs transfected with the Ctnnb1^K49R^ mutant (Fig. 2E). Furthermore, to assess whether expression of the Ctnnb1^K49R^ mutant facilitates the transition from naïve to primed states, we generated ESCs in which endogenous Ctnnb1 was knocked out by CRISPR-Cas9 (Fig. S2) and overexpressed Ctnnb1^K49R^. Upon transfer to Fgf2-supplemented medium, these ESCs formed flat-shaped colonies within 96 h. In addition, the appearance of SSEA1-negative and CD90-positive cells indicated a shift toward an EpiSC-like identity (Fig. S3). As a reciprocal experiment, we generated EpiSCs in which endogenous Ctnnb1 was knocked out and compensated with the Ctnnb1^K49Q^ mutant. To monitor the transition toward the naïve state, we utilized EpiSCs derived from Nanog-green fluorescent protein (GFP) transgenic mice, in which GFP is specifically expressed in the naïve state. Upon Fgf2 deprivation, these Ctnnb1^null/K49Q^ EpiSCs exhibited an elevated expression of naïve pluripotency markers, including Nanog, Klf2, and Klf4, and a subset of cells activated the Nanog-GFP reporter (Fig. S4). These findings suggest that the interaction between K49-deacetylated Ctnnb1 and Nanog observed in primed state interferes with the transcriptional repressor function of Nanog on differentiation-associated genes. To further examine this, we expressed the three Ctnnb1 mutants in ESCs and assessed whether the interaction between Nanog and HDAC2, a core component of the repressor complex, was affected. Consistent with this hypothesis, IP analysis revealed that the expression of the K49R mutant markedly reduced the interaction between Nanog and HDAC2 (Fig. 2F).

### Acetylation of K49 on Ctnnb1 is regulated through differential utilization of the transcriptional coactivators Crebbp and Ep300

**Fig. 3.**
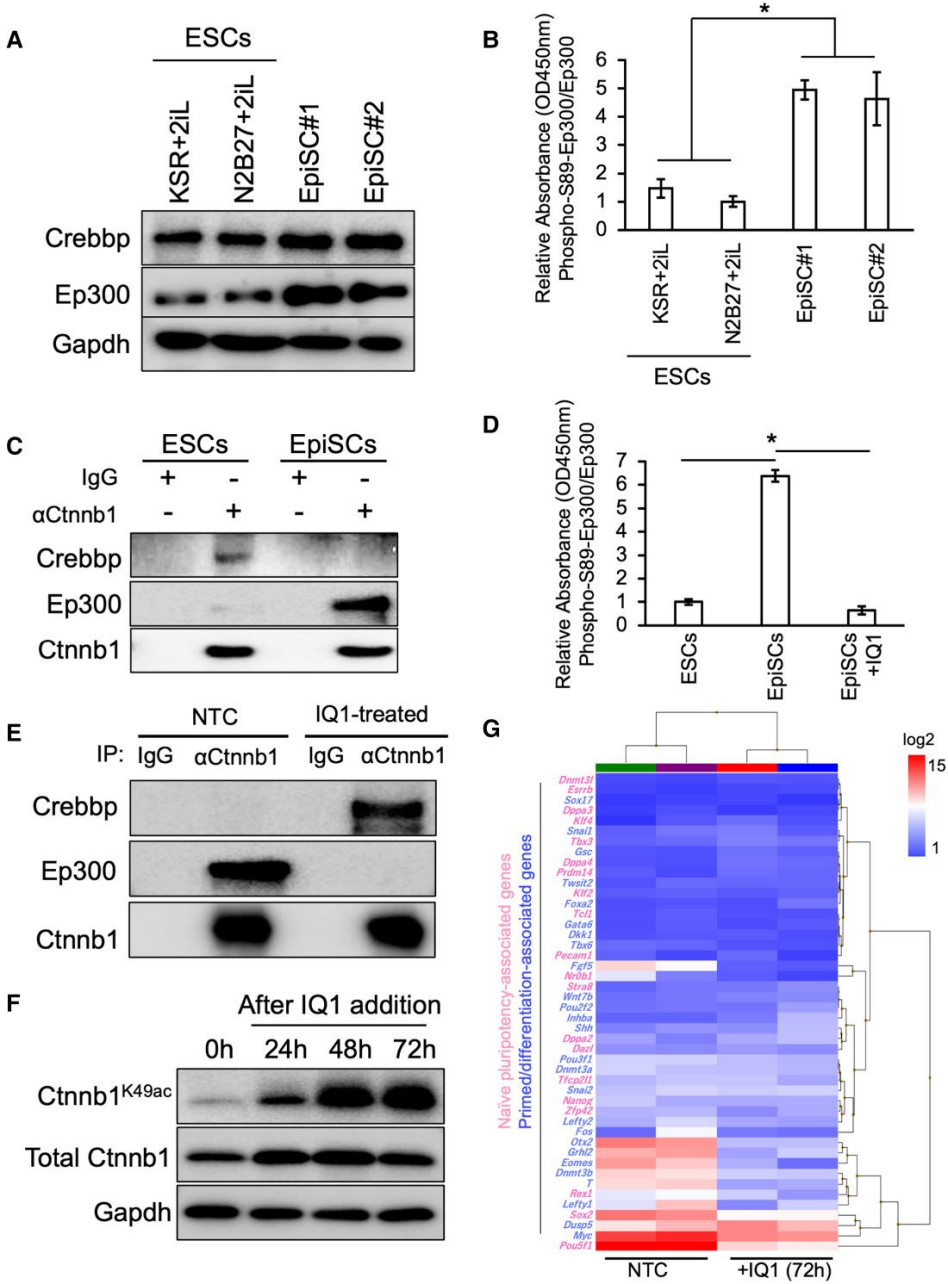
Ctnnb1^K49ac^ is regulated by selective interaction with the histone acetyltransferases (HATs) Crebbp and Ep300; the inhibition of Ep300 function by IQ1 induces naïve-like characteristics in EpiSCs. A) WB showing Crebbp and Ep300 expression levels in ESCs and EpiSCs. B) Quantification of Ep300 S89 phosphorylation in ESCs and EpiSCs using ELISA. The bars represent the mean ± SD of three biological replicates (*n* = 3). The asterisks indicate statistically significant differences between groups (*P* < 0.05). C) IP assays showing Ctnnb1 binding to Crebbp and Ep300 in ESCs and EpiSCs. D) Reduction of Ep300 S89 phosphorylation following IQ1 treatment. The elevated level of Ep300 S89 phosphorylation observed in EpiSCs was significantly decreased upon IQ1 treatment for 72 h. Each bar represents the mean ± SD of three biological replicates (*n* = 3). The asterisks indicate statistically significant differences (*P* < 0.05). E) IQ1-induced shift in Ctnnb1 binding partners in EpiSCs. IP with anti-Ctnnb1 antibody performed 72 h after IQ1 treatment in EpiSCs revealed a preferential enrichment of Crebbp over Ep300. F) WB showing that IQ1 treatment enhances Ctnnb1^K49ac^ in EpiSCs over time. G) Microarray-based gene expression analysis of EpiSCs treated with IQ1 for 72 h. Independent experiments using two distinct cell lines were performed. IQ1 treatment alters the expression profile of signature genes associated with the naïve state (highlighted in red) and primed state/early differentiation (highlighted in blue), inducing naïve-like transcriptional characteristics.

To elucidate the mechanism regulating K49 acetylation on Ctnnb1 in the naïve state, we investigated the roles of the acetyltransferases Crebbp and Ep300 both in naïve and in primed pluripotent states. Previous studies have suggested that Ctnnb1/Crebbp supports the maintenance of the undifferentiated, proliferating state, whereas a shift toward Ctnnb1/Ep300 interaction is associated with the initiating differentiation (24). We therefore measured the expression of Crebbp and Ep300 in ESCs cultured under two distinct conditions: 20% knockout serum replacement (KSR) with PD0325901 and CHIR99021; N2B27 with PD0325901 and CHIR99021, as well as in two independent EpiSC lines under Fgf2 supplemented standard culture condition. We found that Crebbp was expressed at comparable levels across all four stem-cell lines. In contrast, Ep300 exhibited a significantly higher expression in EpiSCs (Fig. 3A).

As the interaction between Ctnnb1 and either Crebbp or Ep300 has been reported to depend on the phosphorylation status of Ep300 at serine 89 residue (S89) (24, 25), we measured the phosphorylation level of Ep300 at S89 by enzyme-linked immunosorbent assay (ELISA) and found it to be ∼5-fold higher in EpiSCs than in ESCs (Fig. 3B). To determine whether Crebbp or Ep300 interacts with Ctnnb1 in ESCs and EpiSCs, both cell types are subjected to IP analysis with an anti-Ctnnb1 antibody, followed by immunoblotting with anti-Crebbp and anti-Ep300 antibodies. The results suggest that Ctnnb1 interacts with Crebbp in ESCs and with Ep300 in EpiSCs (Fig. 3C). Next, we investigated whether IQ1, a compound that can inhibit the phosphorylation of Ep300 (24, 26, 27), can promote the interaction between Crebbp and Ctnnb1 in EpiSCs. Consistent with the reported mechanism, IQ1 treatment markedly reduced the phosphorylation level of Ep300 at S89 in EpiSCs (Fig. 3D). Furthermore, IP analysis revealed that IQ1 treatment suppressed the interaction between Ctnnb1 and Ep300, while concurrently promoting the interaction between Ctnnb1 and Crebbp (Fig. 3E). Consistently, IQ1 treatment results in a significant increase in Ctnnb1^K49ac^ at least after 48 h of treatment (Fig. 3F). When observed at 72 h of IQ1 treatment, the expression of differentiation-related genes including *Fgf5*, *Otx2*, *Eomes*, *Dnmt3b*, and *T* were down-regulated, whereas the expression of transcription factors associated with naïve-state pluripotency, including *Tbx3*, *Stra8*, *Dnmt3l*, *Esrrb*, and *Dppa2*, were slightly up-regulated (Fig. 3G).

### IQ1 treatment promotes naïve pluripotency in the absence of LIF and facilitating primed- to naïve-state conversion

**Fig. 4.**
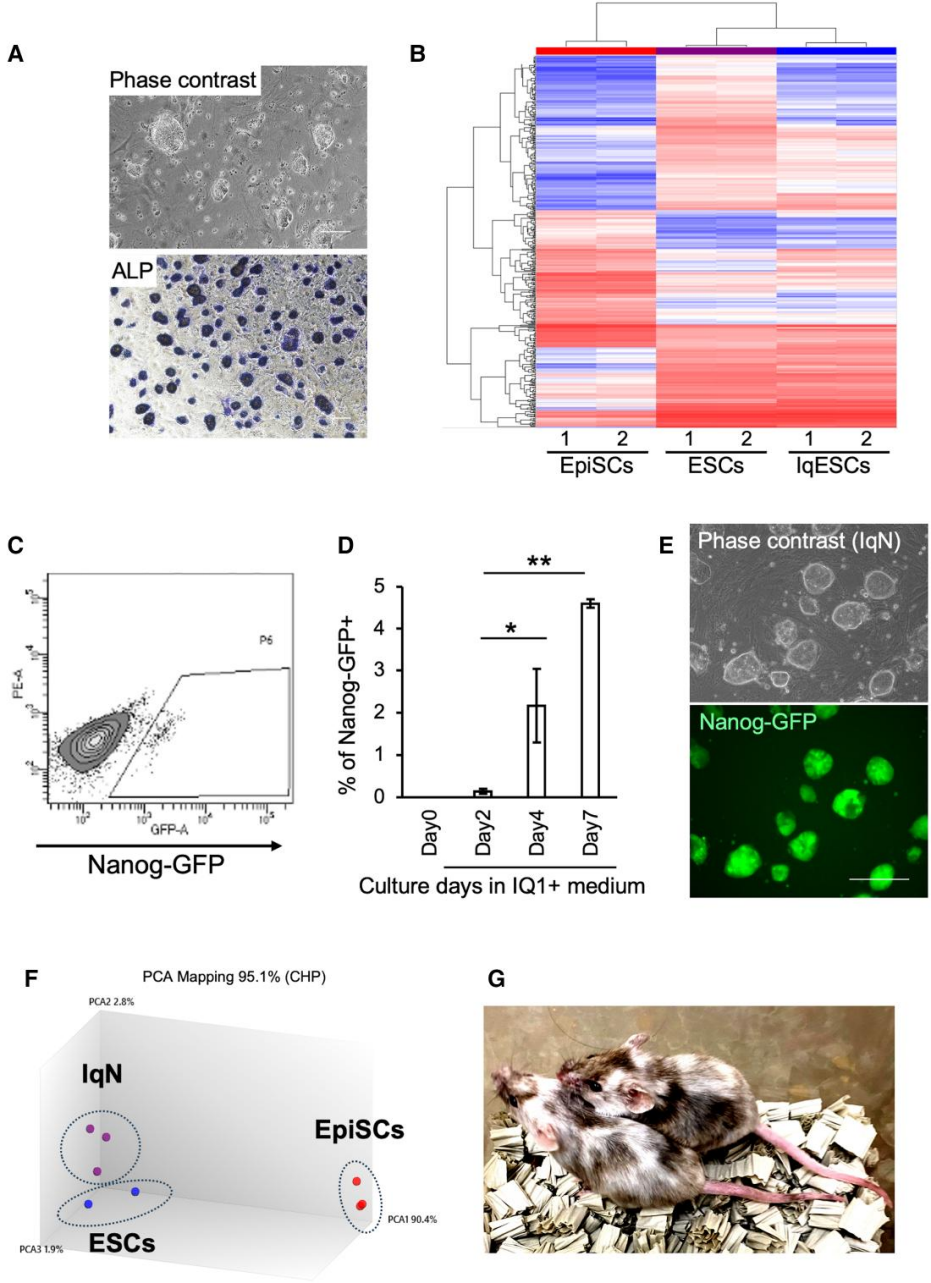
IQ1 enables the self-renewal of ESCs in the absence of LIF and triggers the conversion of EpiSCs to a naïve pluripotent state. A) Phase-contrast images of ESCs established by IQ1 treatment in the absence of LIF. Lower panel, alkaline phosphatase activity staining. B) Transcriptomic analyses on EpiSCs, ESCs, and IqESCs, that is established in IQ1+/LIF-condition. IqESCs exhibit expression profiles similar to those of ESCs. C) EpiSCs cultured in medium supplemented with IQ1 for 4 days began to express Nanog-GFP, as assessed by flow cytometry. D) Progressive increase in Nanog-GFP–positive cells during continuous culture in IQ1-supplemented medium. The bars represent the mean ± SD of three biological replicates (*n* = 3). The asterisks indicate statistically significant differences between groups (*P* < 0.05). E) EpiSCs maintained in IQ1-supplemented medium retain a domed colony morphology with strong Nanog-GFP expression, characteristics of typical naïve-state stem cells. F) Principal component analysis (PCA) of transcriptomic profiles of IqN cells, ESCs, and EpiSCs, showing that IqN cells and ESCs exhibit similar gene expression characteristics. G) Generation of chimeric mice from IqN cells via microinjection into ICR host embryos. As the parental EpiSCs of IqN cells were derived from C57BL/6J mice, the appearance of black fur in chimeric mice serves as an indicator of IqN cell contribution.

Next, we investigated whether the induction of Ctnnb1^K49ac^ by IQ1 treatment is sufficient to establish naïve pluripotency, through two independent experimental approaches. First, to determine the effect of IQ1 in the absence of LIF, we cultured ESCs derived from Oct4ΔPE-GFP Tg embryos, which exhibit high GFP expression in the naïve state, in N2B27 medium supplemented with IQ1. These conditions enabled the propagation of ESCs, while maintaining GFP expression in the absence of LIF, although proliferation was slightly decreased relative to that in the presence of LIF (Fig. S5). Second, we produced ESCs from wild-type murine embryos by culturing them in IQ1-supplemented medium in the absence of LIF. Because embryo adhesion is extremely low in N2B27 medium without LIF and murine embryonic fibroblast (MEF) feeder cells were used to enhance attachment. By day 10 of culture, typical ESC colonies emerged in 20% KSR containing Dulbecco's modified Eagle medium (DMEM) supplemented with 4 µg/mL IQ1, and a stable ESC line was established using serial passaging in IQ1-supplemented medium. This cell line (IqESC) proliferated, maintaining a typical domed colony morphology, and exhibited characteristics associated with the naïve state. Global gene expression analysis revealed that although IqESC displayed slight differences from conventional ESCs, their expression profile was very different from that of the primed state, consistent with the naïve pluripotent state (Fig. 4A and B).

### EpiSCs converted to the naïve state using IQ1 exhibit inner cell mass integration and contribute to chimeras

Finally, we determined whether continuous treatment with IQ1 can convert EpiSCs to the naïve state. When EpiSCs derived from Nanog-GFP transgenic mice, which express GFP exclusively in naïve-state stem cells, were cultured in N2B27 medium containing IQ1, GFP-positive cells were detected at 96 h and increased afterward (Figs. 4C and D and S6). By day 14 of IQ1 treatment, after three passages, all colonies exhibited a domed morphology with strong GFP expression (Fig. 4E). Microarray analysis showed that the IQ1-treated cells had an expression profile highly similar to that of ESCs (Fig. 4F). To maintain clarity in cell-type nomenclature, we refer to these IQ1-treated EpiSCs as IQ1-induced naïve-state pluripotent stem cells (IqNs). To determine whether IqNs possess properties as naïve-state pluripotent stem cells, they were injected into mouse blastocysts after expansion in LIF-supplemented conventional ESC medium without further IQ1 treatment. Consistent with previous reports, control EpiSCs were not observed to integrate into the inner cell mass (ICM) of blastocysts (Fig. S7). In contrast, IqN cells integrated into the ICM of the host embryo and made significant contributions to the resulting chimeric mice (Fig. 4G).

## Discussion

The activity of Ctnnb1 is regulated by multiple posttranslational modifications (PTMs), including phosphorylation, methylation, ubiquitination, and acetylation (28–32). Among these, acetylation of lysine residues by acetyltransferases is thought to be important in modulating Ctnnb1 function (29, 32–35). In this study, we demonstrated that the acetylation status of K49 on Ctnnb1 regulates gene expression through its interaction with Nanog.

In the naïve state, Nanog represses the expression of early lineage–commitment genes, such as *T*, *Cdx2*, *Foxa2*, and *Fgf5*, through Nanog and Oct4-associated deacetylase (NODE) complex with HDACs (36). Additionally, the interaction between Nanog and the Sin3A–HDAC2 complex has been reported to play a critical role in the initiation of reprogramming and the establishment of the naïve pluripotency (37). We observed that K49 of Ctnnb1 was deacetylated in EpiSCs, during which Ctnnb1 bound to Nanog and interfered in its function. IP and BRET analysis using K49 deacetylated mimic Ctnnb1^K49R^ and K49 acetylated mimic Ctnnb1^K49Q^ revealed that K49 acetylation does not affect interaction between Ctnnb1 and Tcf3, which is a well-known partner in the Wnt-Ctnnb1 canonical pathway, but has a critical impact on its interaction with Nanog. In ESCs expressing Ctnnb1^K49R^, early differentiation–associated genes were up-regulated, and the interaction between Nanog and HDAC2 was reduced. These results indicate that deacetylation of Ctnnb1^K49^ impairs the ability of Nanog as a transcriptional repressor. Conversely, the expression of Ctnnb1^K49Q^ induced naïve-like characteristics even in the absence of exogenous supplementation in EpiSCs. These findings are notable because, although Nanog is expressed in EpiSCs, its level is lower than in naïve pluripotent stem cells (38, 39), and its activity appears to be suppressed, as indicated by the expression of differentiation genes. In our study, Nanog was detectable by WB but remained below the detection threshold of the BAC-based Nanog-GFP reporter. The introduction of Ctnnb1^K49ac^ appears to restore Nanog activity, leading to the suppression of early differentiation genes, some of which also act as repressors of Nanog expression. This likely establishes a positive feedback loop that progressively enhances Nanog expression, ultimately leading to the activation of the Nanog-GFP reporter. These results may help explain why Nanog expression remains repressed in the primed state.

As a mechanism regulating Ctnnb1^K49ac^ in pluripotent stem cells, we demonstrated that the preferential interaction between Crebbp and Ctnnb1 plays a critical role in inducing K49 acetylation and subsequently stabilizing the naïve pluripotent state. To date, it has been demonstrated that K49 is the sole lysine in Ctnnb1 acetylated by the histone acetyltransferase Crebbp (34). Although the deletion of either Crebbp or its paralogue Ep300, which shares overlapping functions with Crebbp, does not affect pluripotency or proliferation in ESCs (40), Ep300-null ESCs display marked defects in differentiation (41, 42), suggesting distinct roles for these two molecules during early development. In line with this, we observed increased Ep300 expression and enhanced interaction with Ep300-Ctnnb1 and elevated phosphorylation at S89 (25) in EpiSCs. These findings suggest that in the primed state, Ctnnb1 preferentially interacts with Ep300 rather than Crebbp.

To promote the interaction between Crebbp and Ctnnb1 in EpiSCs, we employed the small molecule IQ1. Mechanistically, IQ1 acts through the inhibition of the PR72/130 regulatory subunit of protein phosphatase 2A (PP2A), thereby regulating the phosphorylation of Ep300 at S89. This phosphorylation event is essential for the Ep300–Ctnnb1 interaction, and its modulation by IQ1 provides a mechanistic basis for redirecting Ctnnb1 toward Crebbp (24, 26, 43, 44). Actually, Rieger et al. (26) have demonstrated that the addition of IQ1 significantly reduces Ep300 S89 phosphorylation in primary alveolar epithelial cells. Consistent with these results, the inhibition of Ep300 by IQ1 promoted K49 acetylation (Fig. S8) as well as a decreased expression of early differentiation genes by the induction of preferential binding between Crebbp and Ctnnb1. Importantly, in Ctnnb1^null^ EpiSCs rescued with a Ctnnb1^K49R^, IQ1 treatment had only a limited effect on suppressing differentiation-associated gene expression, and Nanog-GFP-positive cells did not emerge during our observation period (Fig. S9). These results suggest that a modification of the K49 residue of Ctnnb1 is required to determine the effect of IQ1 on the regulation of pluripotency. Furthermore, these findings also suggest that the interaction partner of Ctnnb1, either Ep300 or Crebbp, acts as a regulatory switch that dictates the maintenance of naïve pluripotency to the initiation of differentiation. These notions may help explain the mechanism underlying the differentiation defects previously observed upon Ep300 deletion (45).

We report that a key effect of IQ1 is likely an induction of Ctnnb1^K49ac^, and that IQ1 treatment alone is sufficient to maintain naïve pluripotency. To date, Miyabayashi et al. (24) already demonstrated that supplementation with IQ1 and Wnt3A allows LIF-independent proliferation of ESCs. While they showed that supplementation with IQ1 alone caused a reduction in ESC proliferation, we observed that naïve characteristics were maintained even under more strict conditions, using N2B27 medium supplemented with only IQ1 (Fig. S5). Additionally, although we cannot exclude the possibility that feeder cells may have functionally compensated for Wnt3A, our findings demonstrate that IQ1 alone is sufficient to establish ESCs. The ESC line established under these conditions, referred to as IqESCs in this study, exhibited global transcriptional profiles that were broadly similar to those of conventional ESCs derived using LIF. Notably, the differentially expressed genes between them did not include naïve-state pluripotency-associated genes (Table S1). These findings suggest that the inhibition of Ep300 and the resulting increase in Ctnnb1^K49ac^ are sufficient to support naïve pluripotency, at least in mice. To further validate this hypothesis, we investigated whether K49 acetylation is sufficient to restore the transcription factor network essential for naïve pluripotency in EpiSCs using IQ1. Unlike ESCs, Nanog-GFP-positive cells derived from EpiSCs proliferated very slowly and required the addition of LIF for stable logarithmic growth. This observation further supports the notion that the functions of Ctnnb1^K49ac^ operate independently of LIF-Stat3 signaling. The naïve-state cells derived from EpiSCs using IQ1 and maintained in LIF supplemented condition, with the cell line being referred to as IqN cells in this study, contributed to tissue and organ formation in chimeric mice. These findings demonstrate that IQ1 supplementation is sufficient to establish naïve pluripotency via a mechanism distinct from previously reported methods for converting primed-state stem cells to the naïve state in mice.

Our findings underscore the significance of Ctnnb1 and highlight the pivotal role of Nanog in establishing naïve-state pluripotency. A previous study has demonstrated that the overexpression of Nanog can drive the conversion of EpiSCs to the naïve state (46). Under such condition, the supraphysiological levels of Nanog might disrupt the quantitative binding equilibrium with Ctnnb1, thereby relieving the inhibitory effect imposed by Ctnnb1 binding in EpiSCs, and enabling Nanog to restore its intrinsic function. Conversely, the up-regulation of early differentiation genes such as *Fgf5* and *Otx2*, resulting from the down-regulation of pluripotency-associated transcription factors including Nanog, further suppress Nanog expression during early development (47, 48). Through this negative feedback between differentiation-associated genes and Nanog, a complete loss of Nanog expression and irreversible differentiation are ultimately established. By contrast, preserving the repressor function of Nanog inhibits the transcription of early differentiation–inducing genes that would otherwise suppress Nanog, thereby maintaining its expression. This shift can initiate a positive feedback loop that supports Nanog transcription, thereby promoting the robust establishment of the naïve state.

On the other hand, Nanog has also been reported to exert bidirectional regulatory effects on Ctnnb1. It interacts with Tcf to inhibit Ctnnb1-mediated transcription (49), while suppressing the expression of *Dkk1*, a negative regulator of the Wnt pathway, thereby indirectly activating Ctnnb1 (50). These findings suggest that Ctnnb1 and Nanog engage in intricate cross talk as key regulators of pluripotency, collaboratively orchestrating both the initiation of differentiation and the maintenance of self-renewal.

## Limitations of this study

This study has at least three limitations. The first is that the detailed molecular mechanisms underlying the positive feedback loop toward the naïve state, triggered by the inhibition of Ctnnb1–Nanog binding in primed stem cells, remain unclear. Because Nanog function is closely linked to important biological processes such as the initiation of differentiation and the regulation of plasticity, its activity is necessarily stringently regulated through interactions with multiple partners, including Ctnnb1, as well as by its own PTMs (51, 52). Our findings demonstrate that nonacetylated Ctnnb1 at K49 suppresses the transcriptional activity of Nanog, and that the induction of this acetylation may be sufficient to activate Nanog function. However, whether this manipulation involves in the binding potential of Nanog to DNA or altered interactions with essential cofactors remains unknown. This directly raises the question whether the control of Ctnnb1^K49ac^ can also induce Nanog expression from more differentiated cells than EpiSCs.

The second limitation is that, although reported cases are limited, Ctnnb1 acetylation has also been observed in other cell types (53, 54), suggesting that this modification may regulate not only Nanog but also the expression of other molecules. For example, K49 acetylation facilitates the nuclear translocation and transcriptional activity of Ctnnb1, leading to the increased expression of the survival-promoting genes Bcl2 and Survivin, which counteract apoptosis. Both are essential regulators of pluripotency with broad functional implications. These molecules may act synergistically to establish the naïve state, and further research is needed to fully elucidate the role of Ctnnb1 in naïve pluripotency.

The third limitation is that it remains unclear whether this phenomenon is reproducible in other species, particularly in humans. Establishing human naïve pluripotency requires more complex steps than in mice and involves a coordinated regulation of multiple signals, such as BRAF, MAPK, GSK3, and PKC, in addition to hLIF and Activin supplementation (55, 56). Ctnnb1 is a highly conserved protein across species, with its PTMs similarly preserved throughout evolution (57). This suggests that Ctnnb1 PTMs likely play a comparable and crucial role in human cells as well. Nonetheless, careful analysis is required to determine their precise impact.

In summary, we demonstrated that K49 acetylation of Ctnnb1 establishes naïve pluripotency by regulating Nanog. This modification can be induced solely by the small molecule IQ1, enabling LIF-independent maintenance of the naïve state and complete conversion from the primed to the naïve state. These findings suggest a molecular mechanism for novel stem-cell manipulations and highlight the versatility of Ctnnb1 and the significance of its physiological functions.

## Materials and methods

### Ethics statement

All procedures related to animal handling, care, surgery, and sacrifice were approved by the Institutional Animal Care and Use Committee of Kindai University as the approved research project number KAME2025-029/031 and performed in accordance with institutional guidelines and regulations. All methods were performed in accordance with ARRIVE guidelines.

### Cell culture

ESCs established from C57BL/6 mice were cultured on gelation-coated dishes or MMC-treated mouse embryonic fibroblast feeder cells in ESC medium, which consisted of Knockout DMEM with MEM nonessential amino acids, 0.1 mM β-mercaptoethanol, 200 mM L-glutamine, and 20% Knockout Serum Replacement (all purchased from Thermo Fisher Scientific, Waltham, MA, United States), supplemented with 1,000 units/mL ESGRO (Millipore, Billerica, MA, United States). EpiSCs established from E5.75 postimplantation embryos were cultured on Matrigel-coated dishes in xeno-free Nutristem XF culture medium (Sartorius, Göttingen, Germany). To passage EpiSCs, 10 µM ROCK inhibitor Y-27632 (FUJIFILM Wako Pure Chemical Corporation, Osaka, Japan) was added to the medium for 24 h after enzymatic digestion to prevent cell death. IQ1 (Selleck Chemicals, Houston, TX, United States), a specific inhibitor of Ctnnb1/Ep300 interaction, was dissolved in DMSO and added to culture medium at a final concentration of 4 μg/mL.

### Plasmid construction and transfection

The pcDNA3-Ctnnb1^S33Y^ plasmid was provided by Addgene (#19286). The pPB-CAG-IRES-Hygromycin and the pPB-CAG-Nanog-IRES-Neomycin plasmids were gifts from Dr Hitoshi Niwa, Kumamoto University, Japan. An HA-tag was added to the N-terminus of Ctnnb1 S33Y, and MluI and NotI recognition sites were added to both ends by PCR. The HA-Ctnnb1 S33Y fragment was subcloned into the MluI/NotI site of the pPB-CAG-IRES-Hygromycin plasmid. For preparation of mutants mimicking the acetylated or nonacetylated forms of Ctnnb1, lysine 49 was replaced with alanine (nonacetylated) or with glutamine (acetylated) by inverse PCR using KOD FX Neo DNA polymerase (Toyobo Co., Ltd, Osaka, Japan). Plasmids were transfected into ESCs or EpiSCs using jetPRIME (Polyplus-transfection, Illkirch-Graffenstaden, France), following the manufacturer's instructions. At 48 h following transfection, cells were collected for quantitative reverse transcription PCR (qPCR) and WB analyses.

### In silico chromatin IP and enrichment

Chromatin immunoprecipitation (ChIP)-sequence analysis was performed with GEO datasets for ChIP-seq public databases for Pou5f1 (SRX214078), Nanog (SRX248286), Sox2 (SRX5023741), and Klf4 (SRX214078). Enrichment analysis was performed using ChIP-Atlas (DBCLS, Tokyo, Japan and Kyusyu University, Fukuoka, Japan) and Integrative Genomics Viewer v. 2.3.90 (Broad Institute, Cambridge, MA, United States).

### BRET assay

To evaluate protein–protein interactions between Ctnnb1 and Nanog, a NanoBRET assay was performed using the NanoBRET Protein:Protein Interaction System (Promega, Madison, WI, United States) in accordance with the manufacturer's instructions. Nanog and Ctnnb1 cDNA were fused with NanoLuc luciferase (donor) and HaloTag (acceptor), respectively, and cloned into the expression vectors. HEK293T cells were seeded into white 96-well plates (Corning Inc., Corning, NY, United States) at a density of 1 × 10^4^ cells per well. The following day, the cells were transfected with donor and acceptor plasmids using Lipofectamine 3000 (Thermo Fisher Scientific). After 24 h, the cells were incubated with HaloTag NanoBRET 618 ligand or DMSO (vehicle control) for 5 h at 37 °C in Opti-MEM medium (Thermo Fisher Scientific). The Nano-Glo substrate was then added, and BRET signals were measured using a TECAN SPARK microplate reader (Tecan Group Ltd, Männedorf, Switzerland) equipped with 460 nm (donor) and 610 nm (acceptor) emission filters.

BRET ratios were calculated as the emission at 610 nm divided by the emission at 460 nm, and background signals from donor-only transfected cells were subtracted. The milli-BRET ratio was calculated using the following formula: {(Acceptor_sample/Donor_sample)−(Acceptor_no tracer/Donor_no tracer)} × 1,000. The experiments were performed in triplicate, and data are presented as mean ± SD from three independent experiments.

### Statistical analysis

All statistical analyses were performed using JMP Pro 18 (SAS Institute, Inc., Cary, NC, United States). Quantitative data, including those from qPCR, flow cytometry, ELISA, and densitometric analysis of immunoblots, were obtained from three independent biological replicates unless otherwise specified. For comparisons among three or more groups, one-way analysis of variance followed by Tukey–Kramer's honestly significant difference post hoc test was performed. For comparisons between two independent groups, Student's t test was used. A *P*-value <0.05 was considered statistically significant.

## Supplementary Material

pgaf297_Supplementary_Data

## Data Availability

Array-based expression profiling data are available in the public database under the following repository name: Characterization of distinct states of mouse naive and primed pluripotency; DOI/accession number(s) GSE107889.
