# Supplementary material for: Acetylation of lysine 49 on Ctnnb1 drives naïve pluripotency in murine stem cells by modulating Nanog function
Source: PNAS Nexus. 2025 Sep 12;4(10):pgaf297. doi: 10.1093/pnasnexus/pgaf297 (PMC12501846; doi:10.1093/pnasnexus/pgaf297)
Supplement: pgaf297_Supplementary_Data [file pgaf297_supplementary_data.pdf]

PNAS nexus

Supplementary Information for

Acetylation of lysine 49 on Ctnnb1 drives naïve pluripotency in murine stem cells by modulating Nanog function

Toshiyuki Takehara[1], Mahito Nakanishi[2,3], Raku Son[4,5,6], Hirofumi Suemori[7], Yasuhiro Murakawa[4,5,8], and Takeshi Teramura[1]

[1]Division of Cell Biology for Regenerative Medicine, Institute of Advanced Clinical Medicine, Kindai University Faculty of Medicine, Osaka, Japan

[2]Tokiwa Bio Inc., Tsukuba, Ibaraki, Japan

[3]Research Center for Stem Cell Engineering, National Institute of Advanced Industrial Science and Technology, Tsukuba, Japan

[4]RIKEN-IFOM Joint Laboratory for Cancer Genomics, RIKEN Center for Integrative Medical Sciences, Yokohama, Japan.

[5]Institute for the Advanced Study of Human Biology, Kyoto University, Kyoto, Japan.

[6]Department of Nephrology, Graduate School of Medicine, Kyoto University, Kyoto, Japan.

[7]Laboratory of Embryonic Stem Cell Research, Institute for Frontier Life and Medical Sciences, Kyoto University

[8]IFOM ETS - the AIRC Institute of Molecular Oncology, Milan, Italy.

Corresponding author: Takeshi Teramura

e-mail: teramura@med.kindai.ac.jp

This PDF file includes:

Supplementary text

Figures S1 to S10

Tables S1 to S3

Supplementary Information

Materials and Methods

Generation of Ctnnb1 knockout EpiSC line using the CRISPR/Cas9 system

To avoid side effects of the truncated RNA, we deleted the coding region of *β-catenin* using two guide RNAs: guide 1, GAGTAGCCATTGTCCACGCAG and guide 2, GCTGGTAAAGCATTTGTGTT. ESC line obtained from a male C57BL/6J mouse embryo was dissociated with TrypLE Express (Thermo Fisher Scientific), washed twice with Opti-MEM (Thermo Fisher Scientific), and re-suspended in 100 μL of Opti-MEM containing 7.5 μg of MLM3636 guide RNA expression plasmid and 5 μg of Cas9 expression plasmid (MLM3636 plasmid: Addgene #43860, and pSpCas9(BB)-2A-Puro (PX459) V2.0: Addgene #62988). The mixture was placed in a transfection cuvette (0.2-cm gap; NEPA gene) and electroporated using a NEPA21 electroporator (NEPA Gene Co., Ltd., Chiba, Japan) with voltage, 150 V; pulse length, 5 ms; pulse interval, 50 ms; number of pulses: 2; decay rate, 10%; polarity, + for poring pulse condition; and voltage, 20 V; pulse length, 50 ms; pulse interval, 50 ms; number of pulses: 5; decay rate, 40%; polarity: +/– for transfer pulse condition. At 24 h after electroporation, 1 μg/μL puromycin was added to the culture medium for 48 h. The transgenic ESCs were cloned and genotyped. For genotyping, the ESCs were digested by KAPA Express Extract (Nippon Genetics Co., Ltd., Tokyo, Japan), diluted in Tris-EDTA and analysed by PCR with KOD FX Neo DNA polymerase (Toyobo Co., Ltd., Osaka, Japan) and genotyping primers listed in Supplementary Table S2.

Immunoprecipitation (IP)

Undifferentiated ESCs, EpiSCs, and Ctnnb1 overexpressed cells were used to IP studies for detection of interaction between Ctnnb1 and pluripotecy-associated transcription factors.

The nuclear fraction was extracted from cells with NE-PER Nuclear and Cytoplasmic Extraction Reagents (Thermo Fisher Scientific), according to the manufacturer's instructions. To isolate CTNNB1 and its associating proteins, the extracts were incubated with anti-HA-tag mAb-magnetic beads (TANA2 clone; MBL Co., Ltd., Nagoya, Japan) or anti-β-catenin pre-treated with Dynabeads (Thermo Fisher Scientific) in 0.02% Tween in TBS buffer at 4°C overnight. Then, the protein complex was magnetically purified, washed three times with 0.02% Tween in TBS buffer, and eluted in SDS buffer for immune blot analysis. The antibodies are listed in Supplementary Table S3.

Immunofluorescence and Alkaline phosphatase assay

To immunofluorescence staining, samples were fixed in 4% formaldehyde solution (Wako) for 30 min, permeabilised in 0.2% Triton-X PBS for 10 min, and treated with 10% Block Ace (Dainippon Sumitomo Pharma Co., Ltd., Osaka, Japan) for 1 h. Then, the specimens were washed twice with 10% Block Ace-PBS and incubated with primary antibody diluted in 10% Block Ace-PBS overnight at 4°C. The samples were washed twice with PBS (–) and reacted with secondary antibody diluted in 10% Block Ace-PBS for 1 h. Prior to fluorescence observation, the samples were counter-stained with DAPI (Vector Laboratories). Images were acquired using a confocal laser microscope equipped with an oil-immersion objective lens (FV-3000; Olympus, Tokyo, Japan). Antibodies and dilution conditions are described in Supplementary Table S3.

Samples were fixed in 4% formaldehyde solution at room temperature for 15 min. Then, the samples were washed three times with 100 mM Tris-HCl (pH 8.2) and treated with alkaline phosphatase substrate (Muto Pure Chemicals Co., Ltd., Tokyo, Japan).

Western blot analysis

Samples were homogenized in SDS buffer (4% SDS, 125 mM Tris–glycine, 10% β-mercaptoethanol, 2% bromophenol blue in 30% glycerol) and centrifuged at 10,000 rcf at 4°C for 10 min to remove debris. Aliquots were subjected to sodium dodecyl sulphate-polyacrylamide gel electrophoresis followed by electrotransfer onto a PVDF membrane (Hybond-P; Cytiva, Tokyo, Japan). The blotted membranes were blocked for 1hr with Block Ace (Dainippon Sumitomo Pharma, Osaka, Japan) or for 10min with 0.1% PVA-TBS and then probed with primary antibody overnight at 4°C. Detection was conducted with horseradish peroxidase (HRP)-conjugated secondary antibodies (all antibodies were purchased from CST) and either the ECL prime Western blotting detection system (Cytiva) or Immunostar® LD (Wako). The immunolabelled membranes were analysed using a CCD-based chemiluminescence analyser (Amersham™ Imager 680; Cytiva). Antibodies and dilution conditions are presented in Supplementary Table S3. Anti-Ctnnb1K49ac antibodies from three manufacturers were evaluated, and the one (BT-AP01077, BT Lab Shanghai Korain Biotech, Shanghai, China) with the lowest background and fewest nonspecific bands was selected (Figure S10).

ELISA

The sample was lysed in RIPA buffer at a concentration of 1 × 10^6 cells per 100 µL, followed by homogenization using a BioRaptor II (Sonicbio Co.,Ltd., Tokyo, Japan) (30 sec on, 30 sec off) for 15 cycles. The lysate was centrifuged at 15,000 rpm for 5 min at 4°C to remove cellular debris. Protein concentration was quantified using a Qubit fluorometer. For ELISA analysis, 1 µg of the sample was used. A total of 100 µL of the sample, diluted to a concentration of 10 µg/mL in 5× ELISA Coating Buffer (BioLegend, San Diego, CA, USA) was added to the ELISA plate (Sumitomo Bakelite Co., Ltd., Tokyo, Japan) and incubated at 37°C for 1 hour to allow solidification. Wells were then blocked with 50% Block Ace/TBS

for 1 hour at room temperature (RT). Subsequently, the primary antibody diluted in 50% Block Ace/TBS was incubated for 1 hour at 37°C. After washing with 0.2% Tween/TBS, HRP-conjugated secondary antibody was added and incubated for 1 hour at 37°C. Following washing, the reaction was developed for 15 min at RT using the ELISA POD Substrate TMB Solution (Hyper) kit (Nakalai Tesque, Tokyo, Japan). Optical density (OD) at 450 nm was measured using the Multiskan G microplate reader (Thermo Fisher Scientific).

In Silico Protein–Protein Interaction Analysis Using HADDOCK

To evaluate the structural interaction between Ctnnb1 and Nanog, an *in silico* protein–protein interaction simulation was performed using HADDOCK (High Ambiguity Driven biomolecular DOCKing). The predicted protein structures of Ctnnb1 (AF-Q02248-F1) and Nanog (AF-Q80Z64-F1) were obtained from the AlphaFold Protein Structure Database. For the K49 acetylated model of Ctnnb1, lysine at position 49 (K49) was substituted with acetylated lysine to enable compatibility with HADDOCK. The active residues for docking were defined as follows: on the Ctnnb1 side, residues 40–55 surrounding the K49 acetylation site were specified as active, and the armadillo repeat region that is known to be involved in protein interactions was also included as an active interface. On the Nanog, a broad range of surface residues were defined as passive residues to allow unbiased interaction modelling. Results were evaluated based on the HADDOCK score, electrostatic interaction energy, and buried surface area (BSA) for each predicted cluster. The model with the highest HADDOCK score was selected for analysis, and its interaction with Nanog was compared to that of the deacetylated form.

Quantitative RT-PCR

Total RNA was isolated using TRI Reagent® (Molecular Research Center, Inc., Cincinnati, OH, USA) and a PrimeScript® RT Master Mix Kit (TAKARA Bio Inc., Shiga, Japan). RNA was reverse transcribed into cDNA using Perfect real-time SYBR green II (Takara Bio Inc). PCRs were conducted in a Thermal Cycler Dice® Real Time System Single at 95°C for 20 s followed by 40 cycles of 95°C for 5 s and 60°C for 30 s. To quantify the relative expression of each gene, the Ct (threshold cycle) values were normalized to those of *Gapdh* ($\Delta Ct = Ct_{target} - Ct_{GAPDH}$) and compared with a calibrator using the$\Delta\Delta Ct$ method ($\Delta\Delta Ct = \Delta Ct_{sample} - \Delta Ct_{control}$). The primer sequences are listed in Supplementary Table S2.

Microarray analysis

RNA probes were prepared and hybridized to an Affymetrix Clariom S microarray for mouse (Thermo Fisher Scientific), following the manufacturer's instructions. The data were analysed by hierarchical clustering with Transcriptome Analysis Console 4.0. (Thermo Fisher Scientific). Statistical significance was evaluated using one-way analysis of variance (ANOVA).

Chimera mouse formation assay

To determine whether IqN contribute to chimera formation as ESCs do, we injected IqN to mouse blastocyst stage embryos. Female ICR mice (8 to 15 weeks old) were super-ovulated by intra-peritoneal injection of 5 IU of pregnant mare serum gonadotropin (1,000 U; Nippon Zenyaku Kogyo) followed 48 h later by 5 IU of chorionic gonadotropin (1,500 U; Nippon Zenyaku Kogyo). The mice were then paired with male ICR mice of proven fertility. Pregnancy was confirmed by the presence of a copulation plug. Two-cell-stage embryos were flushed from the oviduct after 24 h of copulation plug confirmation using M2 medium (Millipore) with 3 mg/mL of bovine serum albumin (Millipore). The collected embryos were cultured in micro-drops of mKSOM (Millipore) covered with mineral oil (Thermo Fisher

Scientific) at 37°C in 5% CO_2 in air. The reconstituted blastocysts were implanted into 2.5-day-pseudopregnant ICR females. Chimeric mouse formation was determined based on the coat colour of the pups.

A

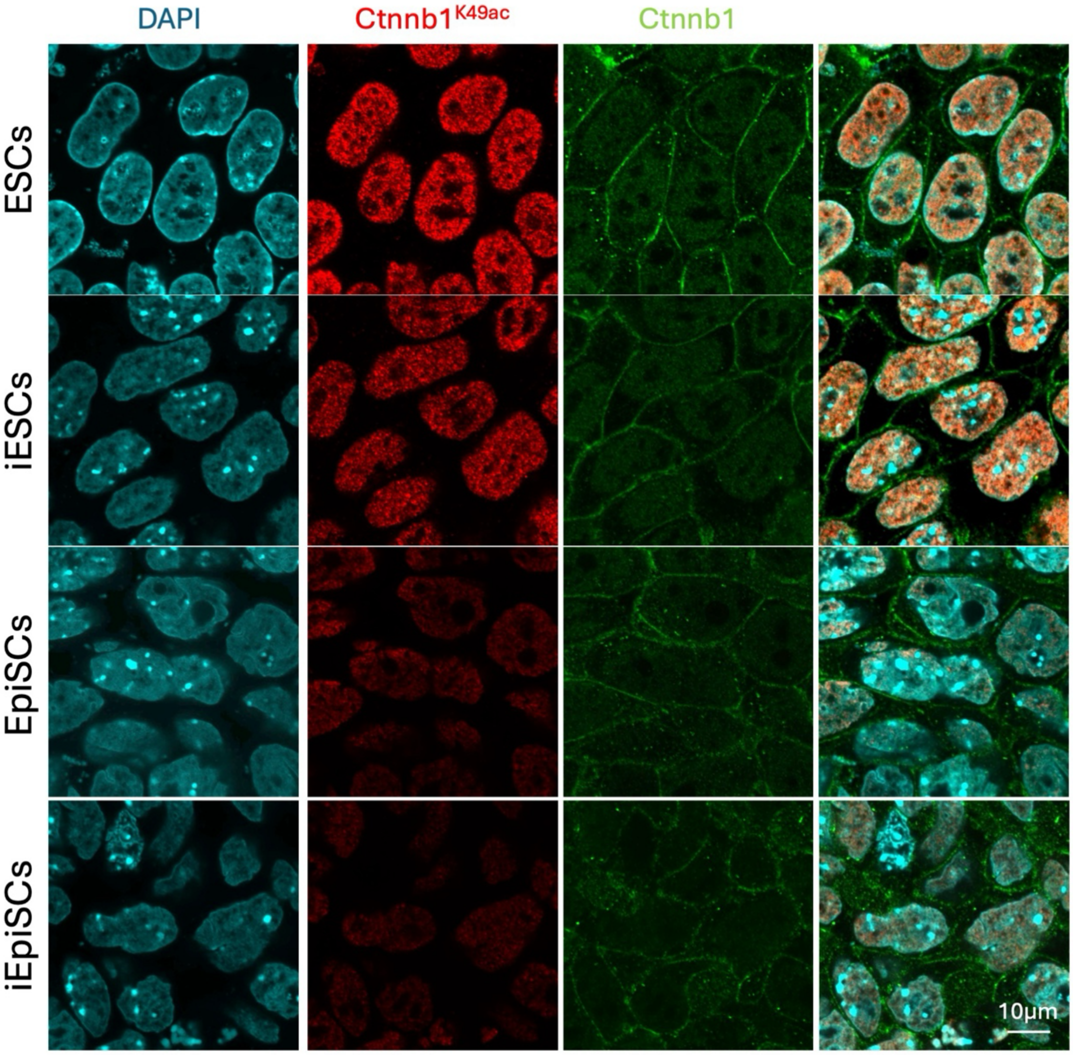

Figure S1. Immunofluorescence of Ctnnb1^{K49ac} in ESCs and EpiSCs. Original, unmerged channel images corresponding to those shown in Figure 1B are provided.

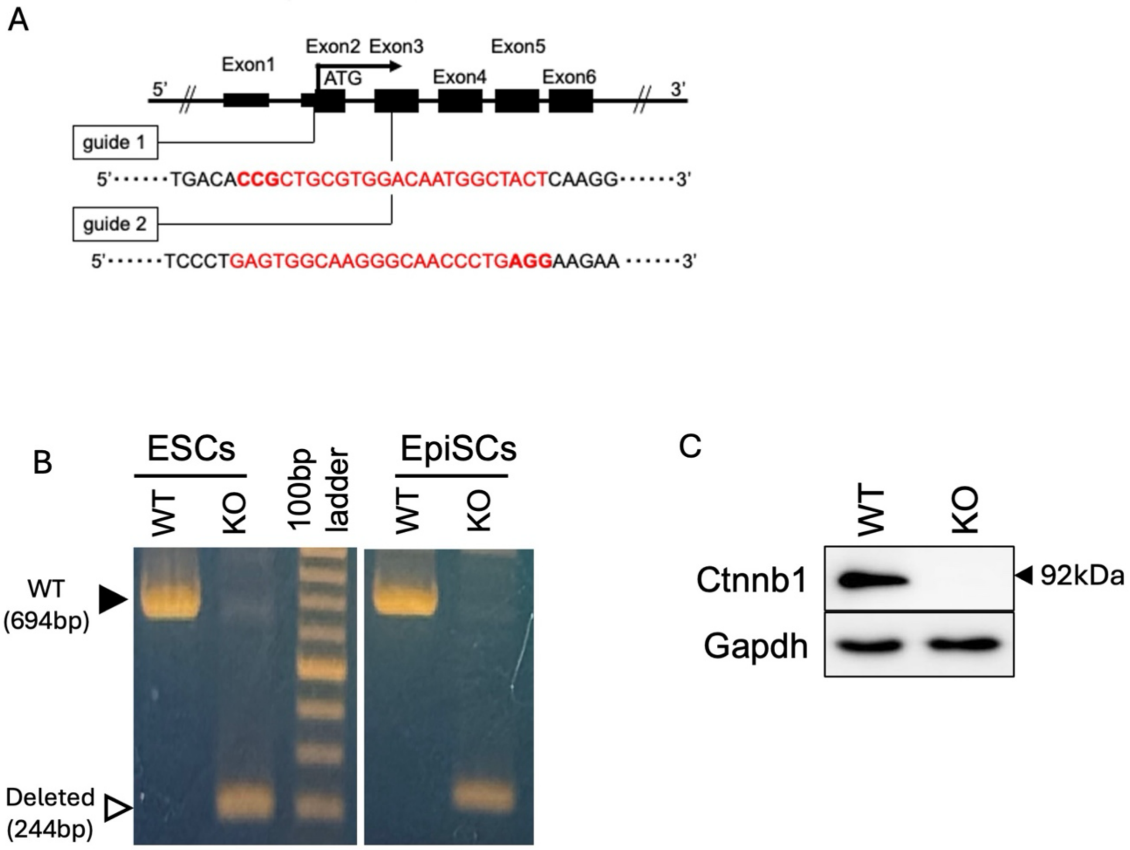

Figure S2. Generation of CRISPR-Cas9-mediated Ctnnb1 knockout ESCs and EpiSCs. (A) gene structure of Ctnnb1 and the guideRNAs recognition sites. (B) genomic PCR showing deletion of Ctnnb1 in the ESCs and EpiSCs. (C) WB analysis to confirm the complete absence of Ctnnb1 in the knockout cells. Data from Ctnnb1^{null} EpiSCs are presented as representative results.

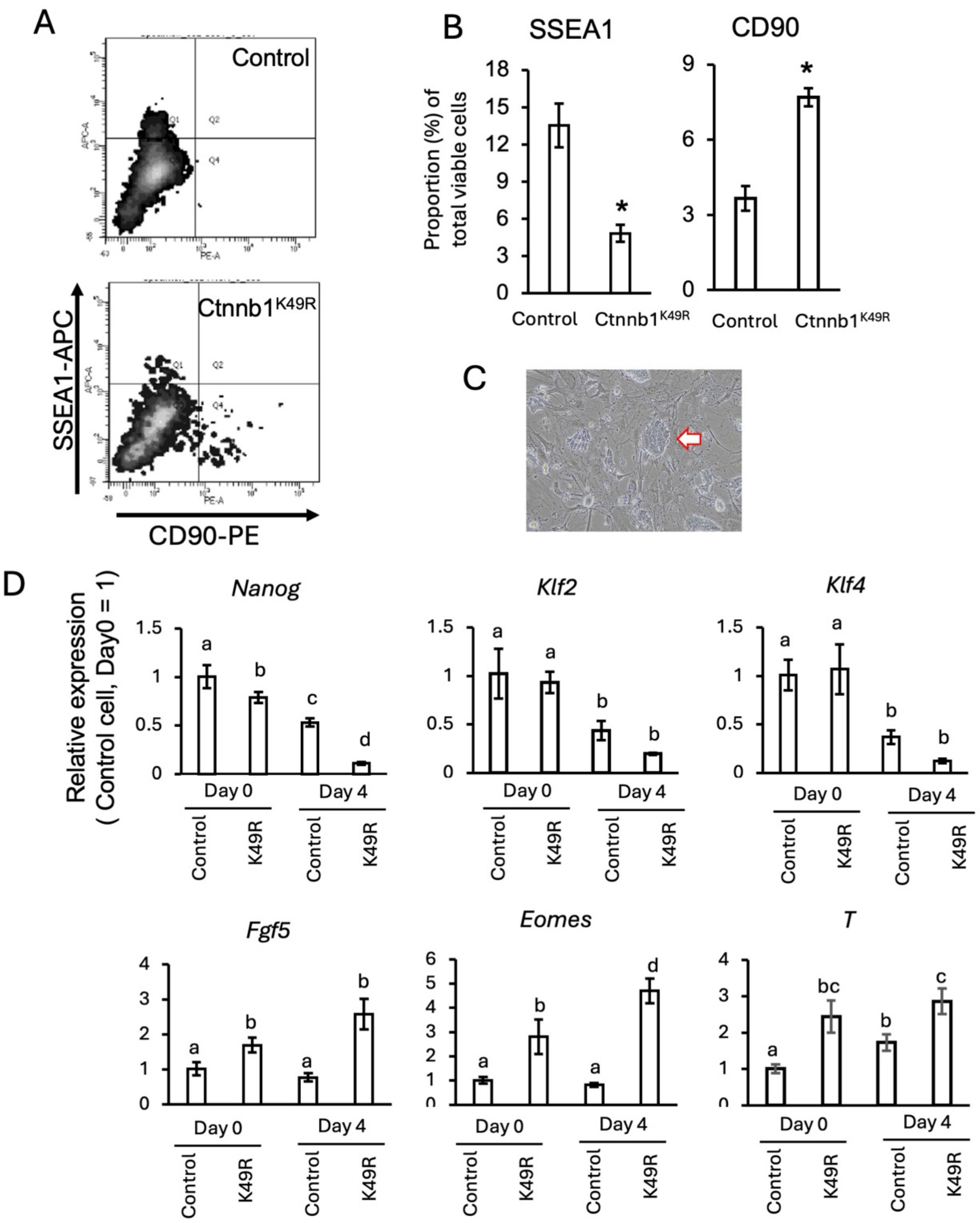

Figure S3. Induction of early differentiation in Ctnnb1^{null} ESCs complemented with Ctnnb1^{K49R} by LIF withdrawal and Fgf2 supplementation. Ctnnb1^{null} ESCs expressing the stabilized Ctnnb1 mutant (Ctnnb1^{S33Y}), that is a parent mutant of Ctnnb1^{K49R}, was used as a control. (A) Flow cytometry analysis of SSEA1 and CD90 expression on day 4 of culture. Naïve cells were identified as SSEA1^{+}/CD90^{-}, whereas primed cells were characterized as SSEA1^{-}/CD90^{+}. (B) Quantification of SSEA1^{+} and CD90^{+} populations on day 4 under LIF-deprived and Fgf2-supplemented conditions. Bars indicate mean ± SD of n = 3 biological replicates. Asterisks denote statistically significant differences between groups ($P < 0.05$). (C) Representative image of EpiSC-like colonies observed on day 4 (arrow). (D) Gene expression changes in control and Ctnnb1^{K49R}-expressing cells. In Ctnnb1^{K49R}-expressing cells, naïve-state markers were markedly downregulated, while the expression of differentiation-associated genes was enhanced.

A

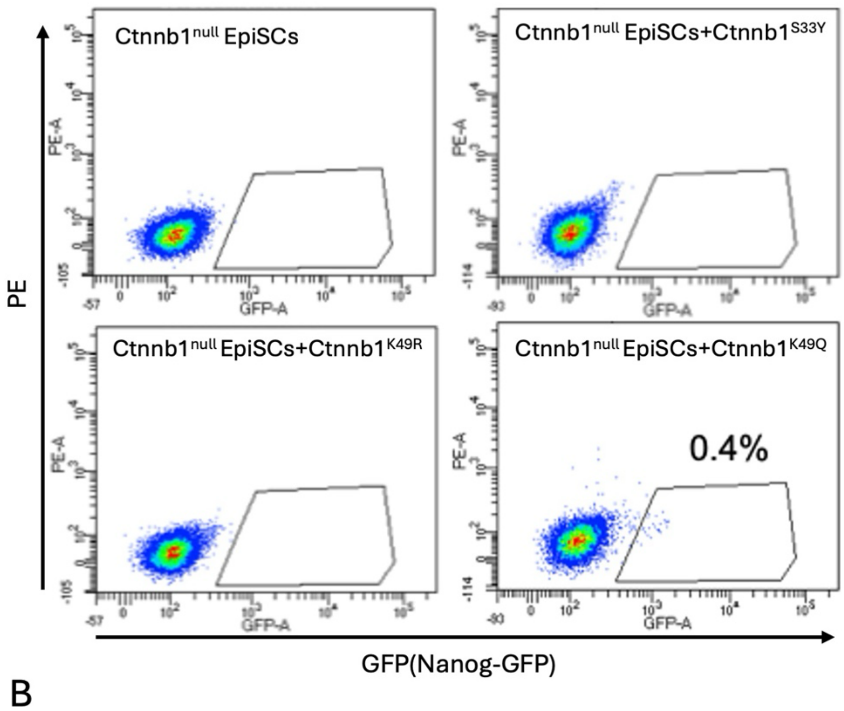

B

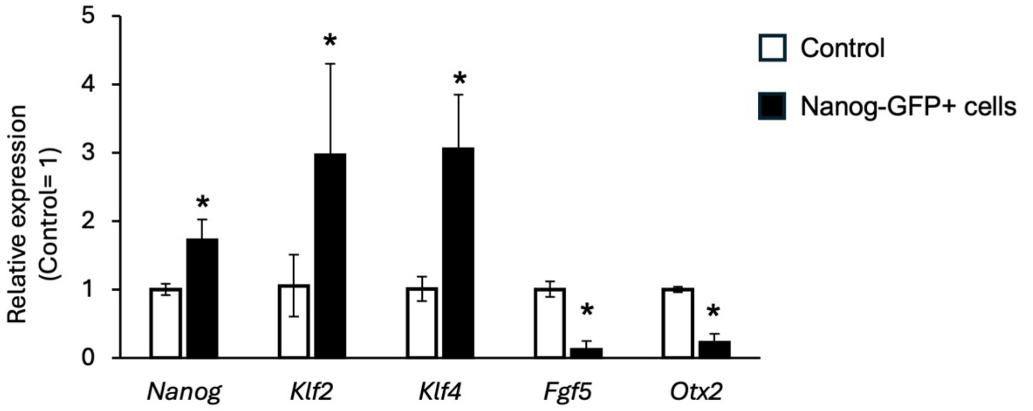

Figure S4. Introduction of plasmids cording stabilized (Ctnnb1^{S33Y}), non-acetylated (Ctnnb1^{K49R}), acetylated (Ctnnb1^{K49Q}) mutants into the Ctnnb1^{null} EpiSCs and monitoring of naïve state transition using Nanog-GFP. (A) Flow cytometry analysis to detect Nanog-GFP expression in Ctnnb1null EpiSCs following the introduction of each Ctnnb1 mutant. (B) qRT-PCR analysis of naïve and early differentiation/primed marker gene expression in Nanog-GFP+ cells that was emerged in the Fgf2-deprived culture condition. For the control group, an equal number of GFP-negative cells was sorted from the same population using FACS. Bars represent the mean ± SD of three biological replicates (n = 3). Asterisks indicate statistically significant differences between groups ($P < 0.05$).

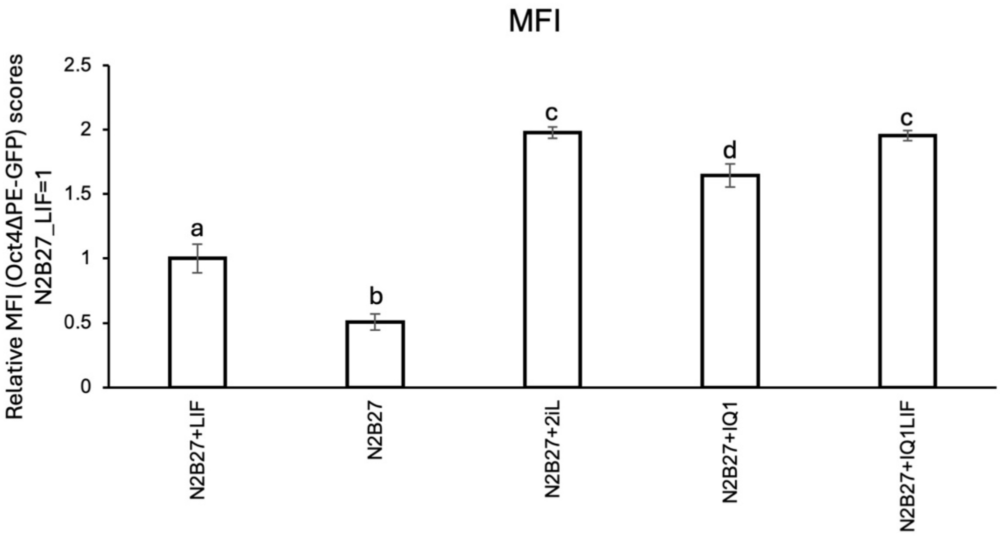

Figure S5. Comparison of expression intensity of Oct4ΔPE-GFP, a marker of naïve pluripotency, in mouse ESCs cultured with LIF or IQ1. In order to eliminate other disturbing factors, the ESCs were cultured on a gelatin-coated surface in N2B27 medium. The 2iL (PD0325901, CHIR99021, and LIF) is described as a control that shows a complete naive pluripotency. Bars represent the mean ± SD of three biological replicates. Different letters above the bars indicate statistically significant differences ($P < 0.05$).

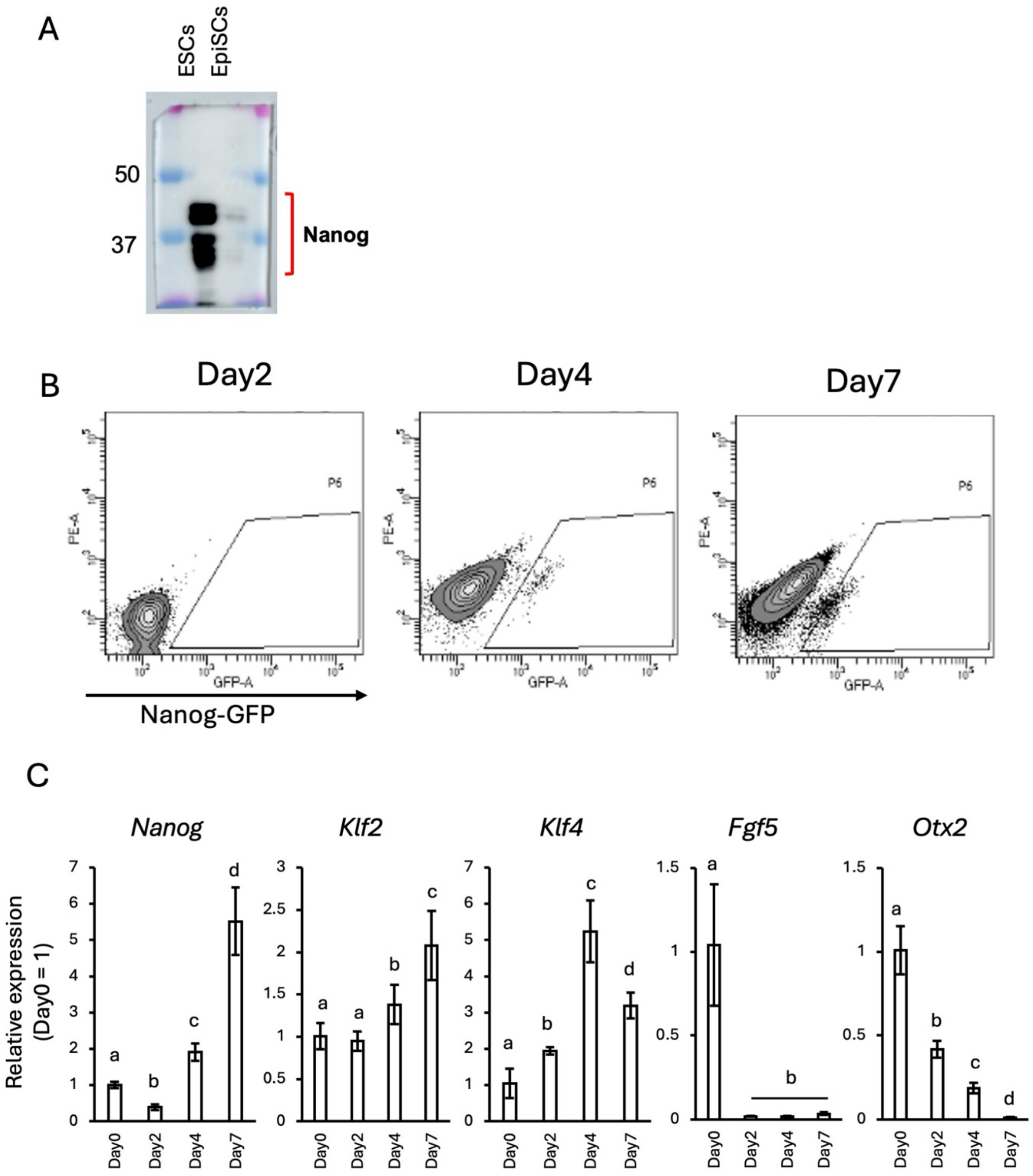

Figure S6. Nanog expression levels in EpiSCs and changes in Nanog-GFP–positive cell populations and gene expression by continuous IQ1 treatment. This figure provides supporting data for Main Figure 4C and 4D. A, Nanog expression in EpiSCs is lower than in ESCs. B, The proportion of Nanog-GFP–positive cells increases progressively over time in EpiSCs cultured with IQ1. C, Gene expression analysis of EpiSCs cultured in IQ1-supplemented medium. Naïve markers were upregulated, while differentiation- and primed-state markers were suppressed. Bars represent mean ± SD. Different lowercase letters indicate statistically significant differences between groups ($P < 0.05$).

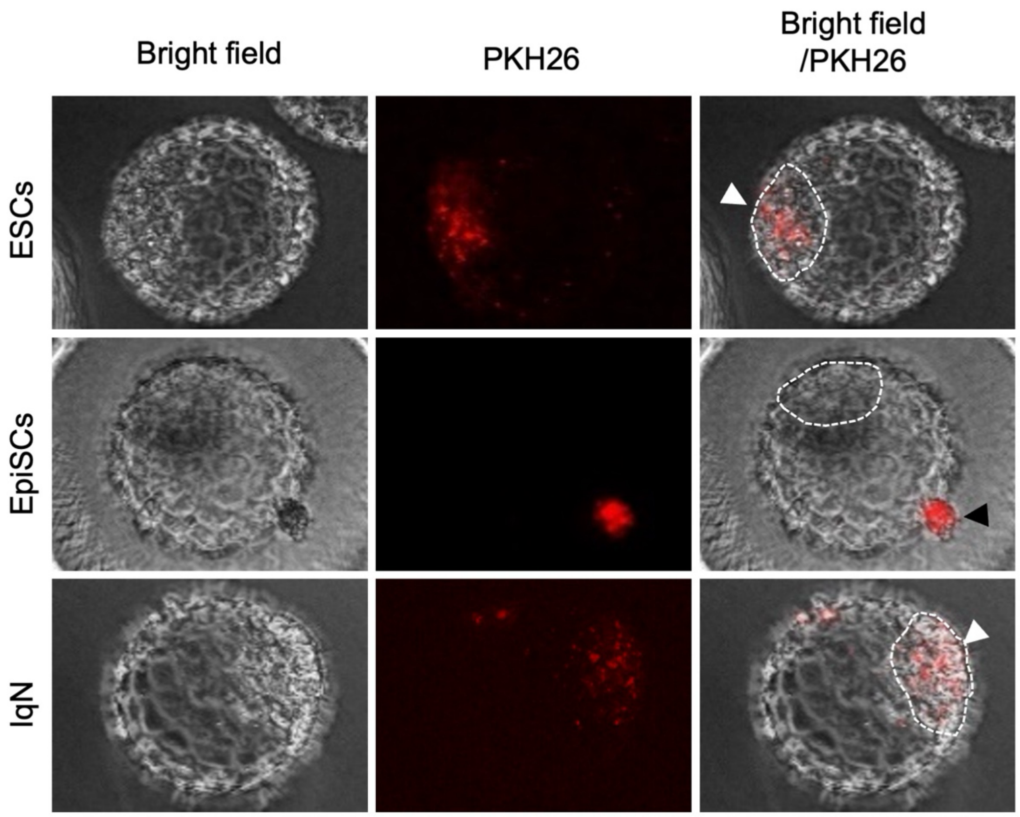

Figure S7. IQ1-induced naïve-state EpiSCs integrated into preimplantation embryos. The top images show integration of ESCs into the inner cell mass (ICM) of the blastocyst. The middle images exclusion of EpiSCs from the embryo. The bottom images show chimeric embryo formation by IQ1-treated EpiSCs.

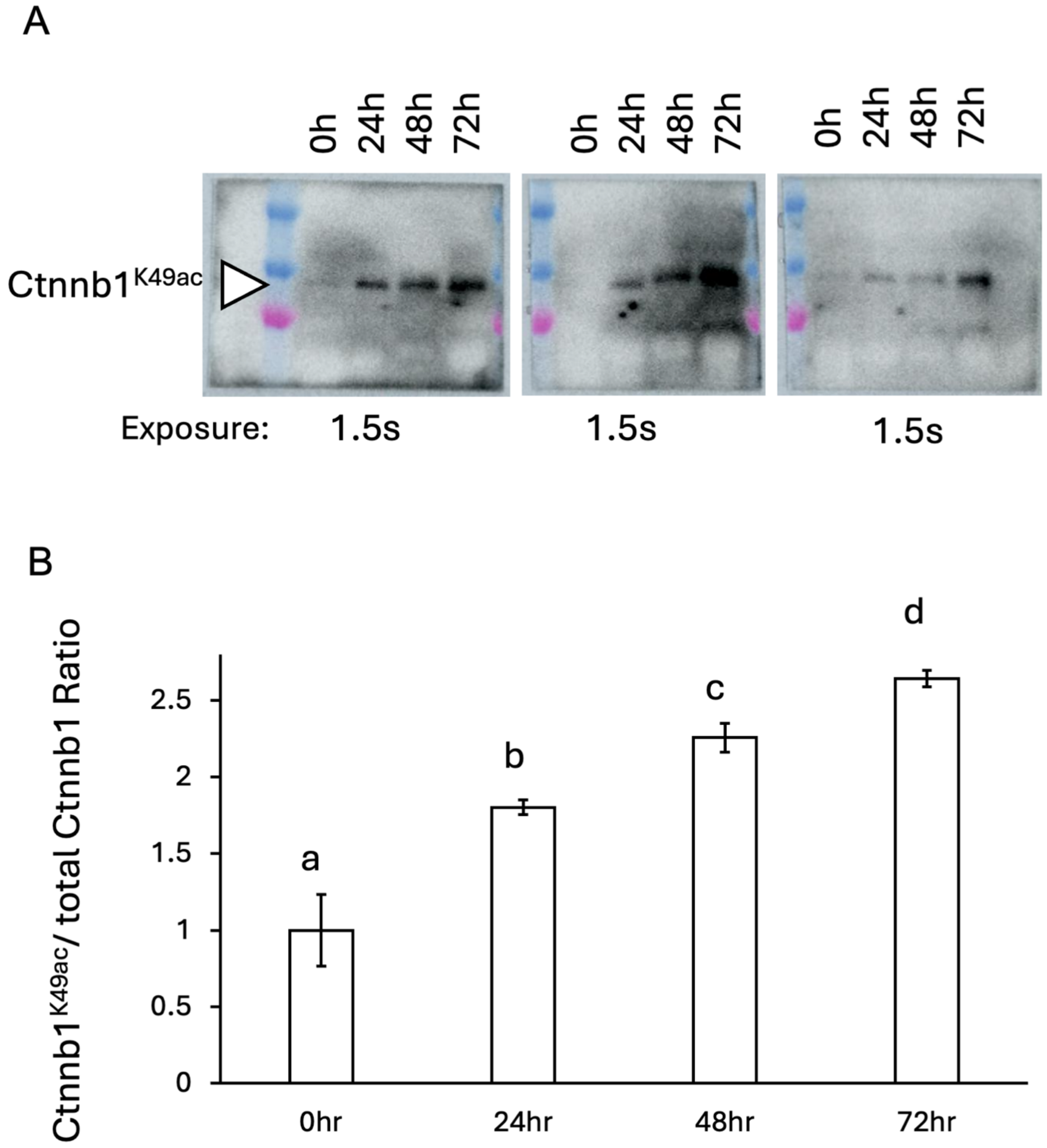

Figure S8. Quantification of Ctnnb1^{K49ac} increase following IQ1 treatment. A, Representative WB images from three independent biological replicates. Unprocessed blot images are shown with exposure times indicated. The arrowhead marks the expected size of Ctnnb1^{K49ac}. B, Semi-quantitative analysis of Ctnnb1^{K49ac} levels normalized to total Ctnnb1 using densitometry. Bars represent the mean ± SD of three biological replicates. Different letters above the bars indicate statistically significant differences ($P < 0.05$).

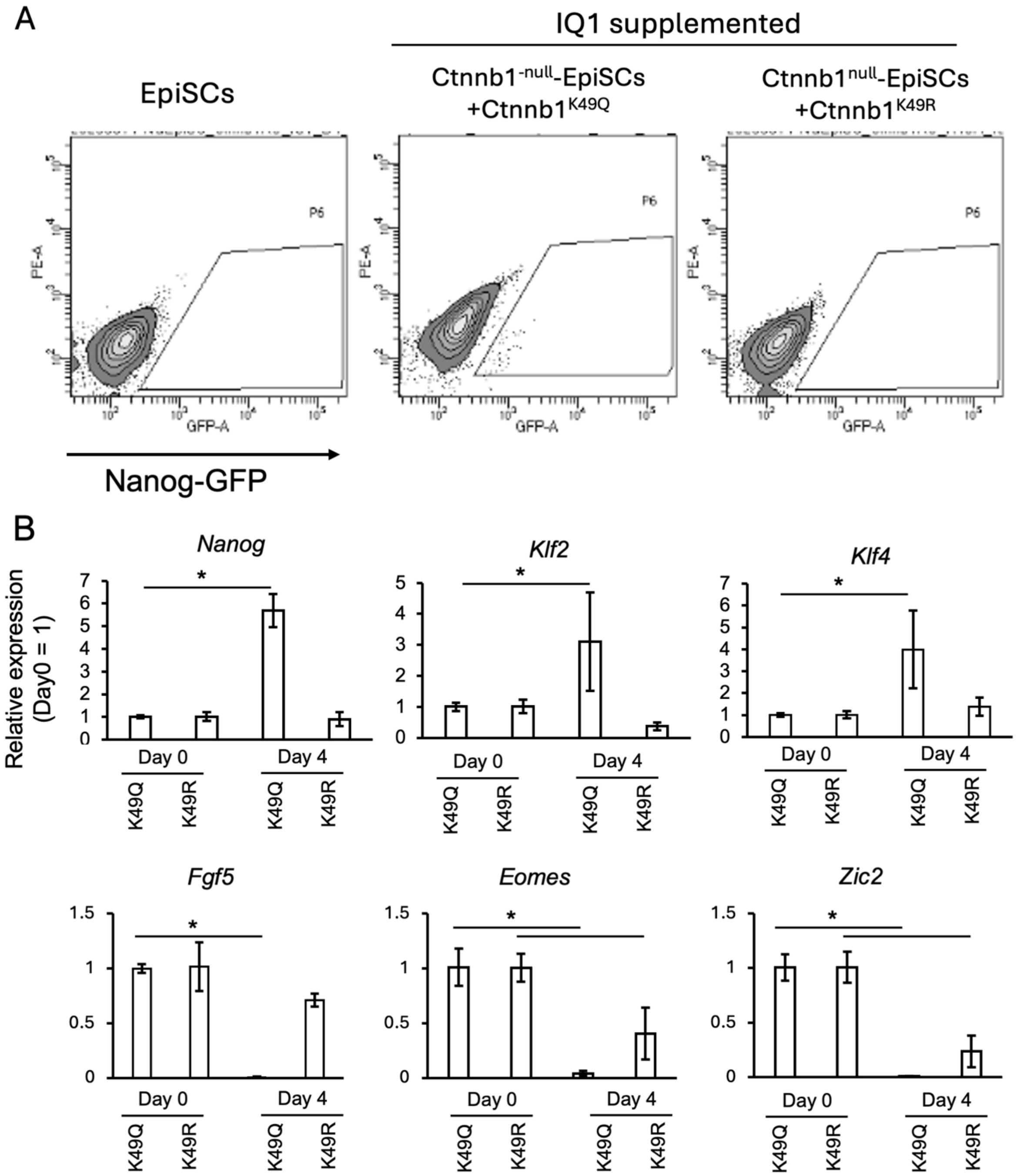

Figure S9. IQ1 treatment in Ctnnb1^{null} EpiSCs complemented with either Ctnnb1^{K49Q} or Ctnnb1^{K49R} mutants. A, Upon Fgf2 deprivation and IQ1 treatment for 4 days, Nanog-GFP expression emerged in the K49Q-expressing EpiSCs but remained undetectable even after 7 days in the K49R-expressing cells. B, Gene expression analysis on day 4 following Fgf2 deprivation and IQ1 addition. K49R-expressing EpiSCs showed no upregulation of naïve pluripotency markers such as *Nanog*, *Klf2*, and *Klf4*. In contrast, the expression of differentiation-associated genes was sustained. Since *Fgf5*, *Eomes*, and *Zic2* are recognized as early differentiation/ EpiSC markers, the reduction in *Eomes* and *Zic2* expression observed in the K49R-expressing cells on day 4 does not necessarily indicate suppression of differentiation.

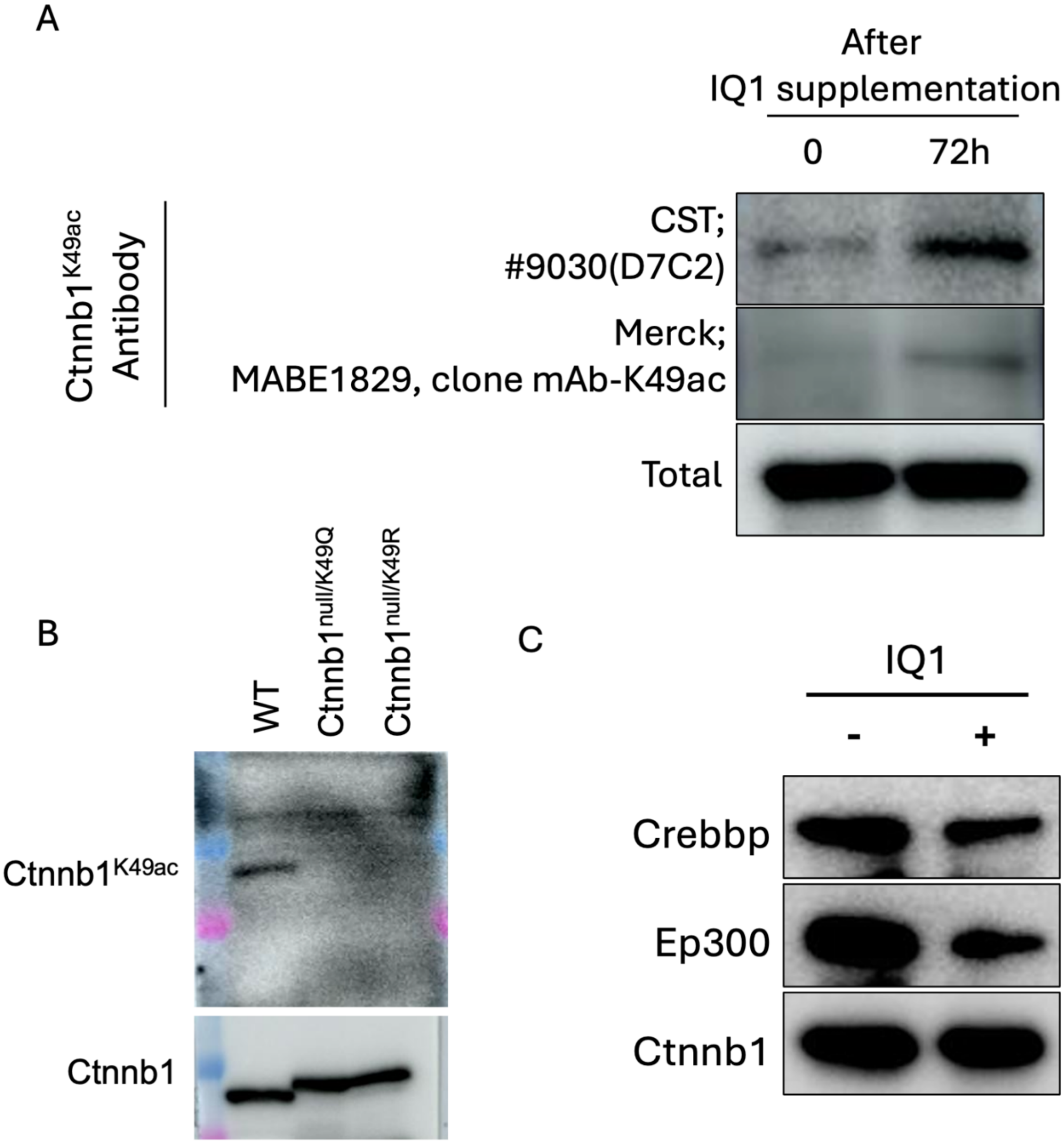

Figure S10. Supporting data for the main experiments. A, Validation of Ctnnb1^{K49ac} upregulation following IQ1 treatment in EpiSCs using two different antibodies. Although the primary antibody (BT-Lab, BT-AP01077) provided the highest S/N ratio in our system (Figure 1 and 3), two additional commercially available antibodies also showed consistent increases in K49 acetylation upon IQ1 treatment. B, WB analysis of Ctnnb1^{K49ac} in ESCs expressing either the Ctnnb1^{K49Q} or Ctnnb1^{K49R} mutant. Each mutant was expressed in ESCs lacking endogenous Ctnnb1 (Ctnnb1^{null}). Immunoblotting using the BT-Lab antibody (employed throughout this study) failed to detect Ctnnb1^{K49ac} even in Ctnnb1$^{null/K49Q}$ ESCs, likely due to conformational alterations caused by the lysine-to-glutamine substitution. C, Input controls corresponding to the IP experiment shown in Figure 3E, confirming the expression of proteins used in the immunoprecipitation.

Table S1. GO terms of differentially expressed transcripts between conventional ESCs and lqESCs.

ID	Source	Term ID	Term Name	p_{adj} (query_1)
1	GO:MF	GO:0005515	protein binding	5.504×10^{-132}
2	GO:MF	GO:0003824	catalytic activity	2.452×10^{-57}
3	GO:MF	GO:0060090	molecular adaptor activity	1.237×10^{-17}
4	GO:MF	GO:0030234	enzyme regulator activity	3.917×10^{-9}
5	GO:MF	GO:0003735	structural constituent of ribosome	8.190×10^{-7}
6	GO:MF	GO:0019843	rRNA binding	4.528×10^{-5}
7	GO:MF	GO:0001228	DNA-binding transcription activator activity, R...	8.623×10^{-5}
8	GO:MF	GO:0005200	structural constituent of cytoskeleton	1.719×10^{-4}
9	GO:MF	GO:0003729	mRNA binding	6.638×10^{-4}
10	GO:MF	GO:0008137	NADH dehydrogenase (ubiquinone) activity	2.876×10^{-3}
11	GO:MF	GO:0140104	molecular carrier activity	9.887×10^{-3}
12	GO:MF	GO:0019783	ubiquitin-like protein peptidase activity	1.517×10^{-2}
13	GO:MF	GO:0001055	RNA polymerase II activity	1.705×10^{-2}
14	GO:MF	GO:0010484	histone H3 acetyltransferase activity	2.695×10^{-2}
15	GO:MF	GO:0051537	2 iron, 2 sulfur cluster binding	3.424×10^{-2}
16	GO:MF	GO:0022804	active transmembrane transporter activity	3.600×10^{-2}
17	GO:BP	GO:0009987	cellular process	6.168×10^{-116}
18	GO:BP	GO:0016032	viral process	4.922×10^{-6}
19	GO:BP	GO:0048511	rhythmic process	1.172×10^{-3}
20	GO:BP	GO:0009410	response to xenobiotic stimulus	1.836×10^{-2}
21	GO:BP	GO:0001678	intracellular glucose homeostasis	1.875×10^{-2}
22	GO:BP	GO:0044403	biological process involved in symbiotic interac...	2.388×10^{-2}
23	GO:BP	GO:0045471	response to ethanol	2.578×10^{-2}
24	GO:BP	GO:0051881	regulation of mitochondrial membrane potential	3.245×10^{-2}
25	GO:CC	GO:0005737	cytoplasm	1.206×10^{-266}
26	GO:CC	GO:0030880	RNA polymerase complex	3.820×10^{-8}
27	GO:CC	GO:0070603	SWI/SNF superfamily-type complex	1.723×10^{-4}
28	GO:CC	GO:0062023	collagen-containing extracellular matrix	2.051×10^{-4}
29	GO:CC	GO:0005681	spliceosomal complex	3.022×10^{-4}
30	GO:CC	GO:0070847	core mediator complex	2.304×10^{-3}
31	GO:CC	GO:0030684	preribosome	3.315×10^{-3}
32	GO:CC	GO:0005652	nuclear lamina	4.283×10^{-3}
33	GO:CC	GO:0016592	mediator complex	1.084×10^{-2}
34	GO:CC	GO:0098562	cytoplasmic side of membrane	1.538×10^{-2}
35	GO:CC	GO:0000801	central element	4.055×10^{-2}
36	GO:CC	GO:0070775	H3 histone acetyltransferase complex	4.055×10^{-2}

Table S2. Primer list used in this study.

Gene	Forward	Reverse
Nanog	ACCAGTGGTTGAAGACTAGCAAT	CTGCAATGGATGCTGGGAT
Klf2	AGACCTACACCAAGAGCTC	TCCCAGTTGCAATGATAAG
Klf4	AGTTCTCATCTCAAGGCACAC	TCACAGTGGTAAGGTTTCTCG
T	ACCAACAAGCTCAATGGAGG	ACGATGTGAATCCGAGGTTC
Fgf5	ATGGCAAAGTCAATGGCTC	TGAGACACAGCAAATATTTCC
Foxa2	AGCCGTGAAGATGGAAG	TGTTCATGCCATTCATCC
Cdx2	ACCTGTGCGAGTGGATG	ACTCCTTCTCCAGCTCCAG
Otx2	AACTTGCCAGAATCCAGG	TGACCTCCATTCTGCTG
Eomes	AAAGCGGACAATAACATGCAG	AGTGGGAGCCAGTGTTAGG
Zic2	TCAAGATCCACAAAAGAACTC	ATGTGCTTCTTCCTGTCG
Gapdh	TCGTGGAGTCTACTGGTGTC	TCGTGGTTCACACCCATCAC
Genotyping for Ctnnb1 KO	TAGCAGAATCACGGTGAC	ACAGCCCTGTCAAGAAAC

Table S3. Antibodies and brief conditions for the experiment.

Application	Antibody (Primary antibody)	Company	Cat No (Clone No)	Dilution	Condition
WB	Mouse monoclonal anti-Oct3/4 Antibody	Santacruz	sc-5279 (C-10)	1/1000 in Immuno-enhancer	4°C, Overnight
WB	Rabbit monoclonal anti-Nanog Antibody	CST	#8822 (D2A3)	1/1000 in Immuno-enhancer	4°C, Overnight
WB	Goat polyclonal anti-Sox2 Antibody	Santacruz	sc-17320 (Y-17)	1/1000 in Immuno-enhancer	4°C, Overnight
WB	Goat polyclonal anti-LKLF(Klf4) Antibody	Santacruz	sc-18690 (N-13)	1/1000 in Immuno-enhancer	4°C, Overnight
WB	Rabbit polyclonal anti-TCF3 Antibody	Santacruz	sc-8635 (M-20)	1/1000 in Immuno-enhancer	4°C, Overnight
WB	Rabbit monoclonal anti-HDAC2 Antibody	CST	#2540	1/1000 in Immuno-enhancer	4°C, Overnight
WB	Rabbit monoclonal anti-CBP Antibody	CST	#7389	1/2500 in Immuno-enhancer	4°C, Overnight
WB	Rabbit monoclonal anti-p300 Antibody	CST	#86377	1/2500 in Immuno-enhancer	4°C, Overnight
WB/IF	Mouse polyclonal anti-βcatenin(14/βcatenin) Antibody	BD Biosciences	610153	WB:1/2000 in Immuno-enhancer/ IF:1/200 in 10% Block-Ace/TBS	4°C, Overnight
WB/IF	Rabbit monoclonal anti-Acetyl-β-catenin(Lys49) antibody	CST	#9030	WB:1/1000 in Immuno-enhancer/ IF:1/200 in 10% Block-Ace/TBS	4°C, Overnight
WB/IF	Rabbit polyclonal anti-Catenin-beta (Acetyl Lys49) antibody	BT LAB	BT-AP01077	WB:1/2000 in Immuno-enhancer/ IF:1/1000 in 10% Block-Ace/TBS	4°C, Overnight
WB	Mouse monoclonal anti-Gapdh antibody	FUJIFILM	014-25524 (5A12)	1 / 2,000 in Immuno-enhancer	4°C, Overnight
WB	Rabbit monoclonal anti-TBP antibody	CST	#8515	1 / 1,000 in Immuno-enhancer	4°C, Overnight
WB	Rabbit polyclonal anti-EP300 antibody	CST	#57625 (E8S2V)	1 / 2,500 in Immuno-enhancer	4°C, Overnight
ELISA	Mouse monoclonal anti-Ep300 antibody	Santacruz	sc-48343 (F-4)	1/500 in 50% Block-Ace/TBS	37°C, 1h
ELISA	Rabbit polyclonal anti-Phospho-Ep300-Ser89 Antibody	St John's Laboratory	STJ91120	1/2500 in in 50% Block-Ace/TBS	37°C, 1h
Wb	Goat Anti-mouse IgG, HRP-linked Anbitody	CST	#7073	1/2000 in Immuno-enhancer	4°C, Overnight
Wb	Goat Anti-rabbit IgG, HRP-linked Anbitody	CST	#7074	1/2000 in Immuno-enhancer	4°C, Overnight
IF	Donkey Anti-IgG(H+L), Rabbit CFTM555	BTI	200038-1MG	1/1000 in 10% Block-Ace/TBS	RT, 1h
IF	Donkey Anti-IgG(H+L), Mouse CFTM488	BTI	200038-1MG	1/1000 in 10% Block-Ace/TBS	RT, 1h
